# Theory of Field-Dependent NMR Shifts in Paramagnetic Molecules

**DOI:** 10.1021/acs.jctc.5c00433

**Published:** 2025-05-27

**Authors:** Lucas Lang, Letizia Fiorucci, Giacomo Parigi, Claudio Luchinat, Enrico Ravera

**Affiliations:** † Theoretische Chemie/Quantenchemie, Institut für Chemie, 26524 Technische Universität Berlin, Sekr. C7, Straße des 17. Juni 135, 10623 Berlin, Germany; ‡ Department of Chemistry “Ugo Schiff” and Magnetic Resonance Center (CERM), University of Florence, 50019 Florence, Italy; § Consorzio Interuniversitario Risonanze Magnetiche di Metalloproteine (CIRMMP), 50019 Florence, Italy; ∥ Giotto Biotech S.R.L, 50019 Sesto Fiorentino, Italy; ⊥ Florence Data Science, 9300 University of Florence, 50134 Florence, Italy

## Abstract

NMR chemical shifts depend on the applied magnetic flux density, and this becomes more and more important as stronger and stronger magnetic fields are becoming available. Herein we develop a theory of the field dependence of NMR shifts of paramagnetic molecules in solution. Our derivation leads to two distinct approaches: a finite-field approach that describes the shift up to infinite order in the applied field *B*_0_ but requires numerical integration for the orientational average, and a second-order approach that is valid up to second order in *B*_0_. In this latter approach, the orientational average can be performed analytically, and the field dependence cleanly separates into two additive terms: the well-known “indirect” field dependence due to incomplete averaging in solution and the “direct” field dependence due to the nonlinear response to the external field. In analogy to the diamagnetic case, the direct field dependence involves a fourth-order tensor **τ** whose elements are fourth derivatives of the electronic Helmholtz free energy. Generalizing the Van den Heuvel–Soncini equation, we provide analytical sum-over-states equations for these higher-order derivatives. Using the NiSAL–HDPT complex as an example, we demonstrate the applicability of the second-order approach at room temperature and the highest commercially available field strength and show that it agrees well with the field dependence measured experimentally.

## Graphical abstract

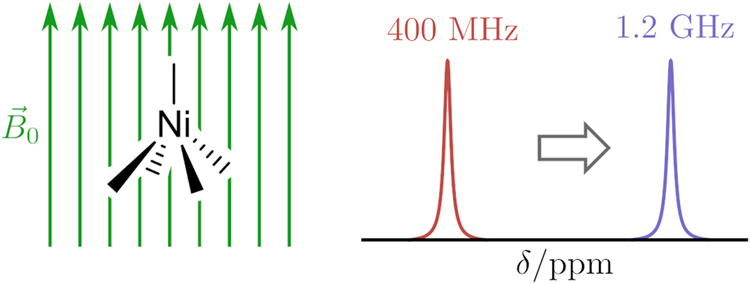

## 1 Introduction

When molecules are placed into an external magnetic field, the response of the electrons leads to an additional induced magnetic field, which gives rise to chemical shifts in NMR spectroscopy. It is usually assumed that the induced field is linear in the external field, with the “proportionality constant” being the chemical shielding tensor **σ**. Under this assumption, chemical shifts are field-independent. For example, one would obtain the same shifts in parts per million (ppm) at 400 or 600 MHz ^1^H Larmor frequency. However, at strong enough fields, nonlinear effects become increasingly important. This becomes relevant as commercially available NMR spectrometers continuously move towards higher magnetic fields (currently up to 1.2 GHz ^1^H Larmor frequency) because of benefits like higher resolution and sensitivity.^1^ In non-commercial contexts, even stronger fields than that have been achieved.^2^

A well-known “indirect”^3^ effect of magnetic field strength on the measured chemical shifts is due to the non-isotropic averaging of the chemical shielding tensor in solution, which is known as residual chemical shift anistropy.^4,5^ The non-isotropic averaging is caused by partial orientation of the molecules with respect to the external field direction, because some orientations of the molecule have a lower energy than others in the case of an anisotropic magnetic susceptibility tensor. Of course, this effect is larger for paramagnetic molecules, which have intrinsically larger anisotropies.^6–9^

On top of this indirect effect, there is the “direct”^3^ field dependence caused by a nonlinear response of the induced field to the external field. For diamagnetic molecules, Ramsey theoretically predicted this phenomenon already in 1970 ^10^ with the introduction of the **τ** tensor, which describes the part of the induced field that is cubic in the external field (the quadratic part always being identically zero). This was then proven by measurements of field-dependent ^59^Co NMR shifts in diamagnetic cobalt­(III) complexes.^11^ Over the years, the effect was theoretically treated by various authors^12–17^ until a recent joint experimental and theoretical study^3^ corrected the old values for the ^59^Co chemical shifts in the Co­(acac)_3_ complex by an order of magnitude, and finally reconciled measurements and theoretical predictions.

For paramagnetic molecules, field-dependent chemical shifts are much less explored, although the magnitude of the effect is much larger. In 1998, Bertini et al. investigated field-dependent shifts in paramagnetic lanthanoid complexes and found that the indirect effect was overcompensated by the direct effect, which is of opposite sign and has a larger magnitude.^18^ In that paper, the authors estimated the direct effect by scaling the susceptibility tensor using the Brillouin equation, which however does not properly take into account the anisotropy of the effect.

Herein, we properly develop the theory of field-dependent solution NMR shifts in paramagnetic molecules, taking into account both indirect and direct effects. It would also be straightforward to extend the theory to the solid state case. It is possible to formulate the theory using the exact induced field (up to infinite order in the external field). However, this requires numerical integration over all possible orientations of the molecule in solution. An alternative is the expansion of the induced field up to third order in the external field *B*_0_, which makes it possible to perform the integration over orientations analytically. This corresponds to shifts that are correct up to second order in *B*_0_. In this context, it turns out that the generalization of Ramsey’s **τ** tensor to paramagnetic molecules can be expressed as a fourth derivative of the Helmholtz free energy. The Van Vleck equation for the susceptibility tensor^19,20^ and the Van den Heuvel–Soncini equation for the chemical shielding tensor^21^ are analytical sum-over-states expressions for second derivatives of the Helmholtz free energy. Analogously, in order to calculate the **τ** tensor, we derive an analytical sum-over-states equation for fourth derivatives of the free energy.

Finally, the new theory is tested and illustrated on a concrete example, the NiSAL–HDPT complex,^22^ using a spin Hamiltonian and *ab initio* ligand field theory (AILFT) parametrization.

## 2 Theory

### 2.1 Average Induced Field

We consider a molecule in solution, allowing for more than one thermally accessible electronic state, as is common for paramagnetic molecules. However, the theory is fully general and is also applicable to diamagnetic molecules as a special case. Due to interactions with the environment in solution, e.g., collisions with other molecules, the molecule can undergo electronic transitions as well as whole-body rotations. Both processes are assumed to occur on a time scale much faster than the time scale of the nuclear spin dynamics, such that the dynamics of the magnetic nucleus can be described with a Hamiltonian that is a time average of the true Hamiltonian. Since the nuclear Zeeman Hamiltonian is linear in the magnetic field *B⃗*, this effect can be described by averaging *B⃗* over all possible thermally accessible electronic states and molecular orientations, weighted with their probability. We treat the electrons quantum-mechanically and the rotations classically, assuming that the molecule is otherwise rigid. This is similar to how paramagnetic molecules are usually modelled in semi-classical relaxation theory.^23^

We make use of two different types of coordinate systems: A laboratory frame, with orthonormal basis vectors *e⃗*_*X*_, *e⃗*_*Y*_, *e⃗*_*Z*_, in which the external field **B**_0_^lab^ is constant, and a molecule-fixed frame, with orthonormal basis vectors *e⃗*_*x*_, *e⃗*_*y*_, *e⃗*_*z*_, which is rotating together with the molecule such that the coordinates of the nuclei expressed in this frame are constant. Orientations of the molecule can then be described by the rotation matrix **R** that transforms the laboratory frame basis into the molecule-fixed basis:

1 $${\mathrm{e⃗}}_{i}=\sum_{I=X,Y,Z}{\mathrm{e⃗}}_{I}R_{Ii},\left(i\in x,y,z\right)$$

For example, **R** = **I** (the unit matrix) means that the laboratory frame and the molecule-fixed frame coincide. The rotation matrix **R** can be parametrized with three Euler angles; see Section 2.2 below. The two frames are illustrated in Figure 1.

**Figure 1:**
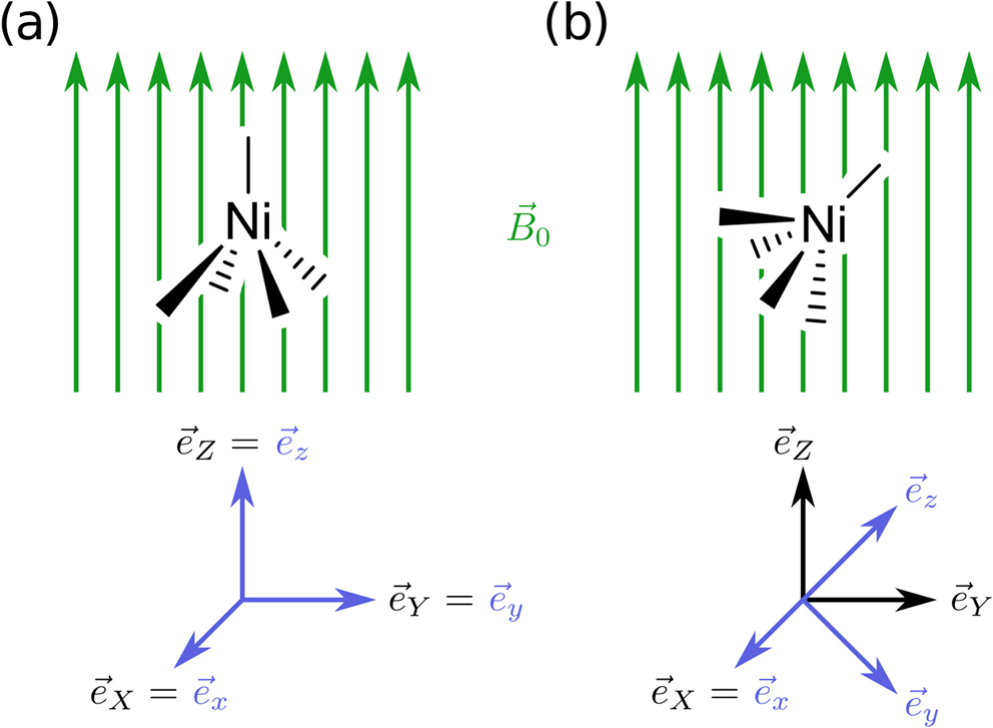
Illustration of the laboratory and molecule-fixed frames for two different orientations of a molecule. The laboratory frame is shown in black and the molecule-fixed frame in blue. It is common to choose the *e⃗*_*Z*_ basis vector of the laboratory frame such that it points in the direction of the external field *B⃗*_0_. The molecule-fixed frame is always aligned with the molecule, i.e., the coordinates of the nuclei in this frame are constant under rotations of the molecule. In (a) the two frames coincide, whereas in (b) the molecule-fixed frame is rotated by −45° around the *X* direction with respect to the laboratory frame. The orientation depicted in (b) corresponds to Euler angles of χ = −90°, θ = 45°, and ϕ = 90° (see Section 2.2 below for a description of the Euler angle convention used herein).

In a particular electronic state *i* and orientation **R** with respect to the static magnetic field, we write *E*(*i*, **R**) for the total electronic energy of the molecule. The probability (density) to find the system in a given microstate in thermodynamic equilibrium is given by the Boltzmann factor

2 $$p\left(i,R\right)=\frac{1}{Z^{\mathrm{tot}}}e^{-\beta E\left(i,R\right)}$$

3 $$Z^{\mathrm{tot}}=\sum_{i}\int e^{-\beta E\left(i,R\right)}dR$$

where *Z* ^tot^ is the canonical partition function and β = 1/*k*_B_ *T*. The exact definition of the integration over rotations **R** is provided in eq 15 below. Hence, the average induced field can be written as

4 $$\langle B^{\mathrm{ind}}\rangle^{\mathrm{tot}}=\sum_{i}\int p\left(i,R\right)B^{\mathrm{ind}}\left(i,R\right)dR$$

with the induced field of a particular microstate given as

5 $$B^{\mathrm{ind}}\left(i,R\right)=-\left\langle i\left(R\right)\left|\frac{\partial H}{\partial M}\right|i\left(R\right)\right\rangle$$

Here, **M** is the nuclear magnetic moment. Note that all vectors in this section (including **B** ^ind^(*i*, **R**)) refer to the laboratory frame. The marginal probability to find the system in a given orientation (with *any* electronic state) can be written as

6 $$p\left(R\right)=\sum_{i}p\left(i,R\right)=\frac{Z^{\mathrm{el}}\left(R\right)}{Z^{\mathrm{tot}}}$$

where we defined the electronic partition function in a given orientation via

7 $$Z^{\mathrm{el}}\left(R\right)=\sum_{i}e^{-\beta E\left(i,R\right)}=\mathrm{tr}\left(e^{-\beta H\left(R\right)}\right)$$

Here, *H*(**R**) is the electronic Hamiltonian whose eigenstates are expressed with respect to the molecule-fixed frame. Its **R**-dependence comes from its dependence on the external magnetic field, which in the molecule-fixed frame depends on the orientation. Equation 6 can be rewritten as 1/*Z* ^tot^ = *p*(**R**)/*Z* ^el^(**R**). Therefore, the average induced field [eq 4] becomes

8 $$\langle B^{\mathrm{ind}}\rangle^{\mathrm{tot}}=\int p\left(R\right)\frac{1}{Z^{\mathrm{el}}\left(R\right)}\sum_{i}e^{-\beta E\left(i,R\right)}B^{\mathrm{ind}}\left(i,R\right)dR=\int p\left(R\right)\langle B^{\mathrm{ind}}\rangle^{\mathrm{el}}\left(R\right)dR=\langle \langle B^{\mathrm{ind}}\rangle^{\mathrm{el}}\left(R\right)\rangle^{\mathrm{rot}}$$

This equation means that we can first average over all electronic states at a given orientation to obtain the induced field Boltzmann-averaged over all thermally populated electronic states in a given orientation, ⟨**B** ^ind^⟩^el^(**R**), and in a second step average over all orientations.

The marginal probabilities *p*(**R**) occurring in eq 8 can be expressed without reference to the microstate energies *E*(*i*, **R**). The numerator and denominator in eq 6 can be written as

9 $$Z^{\mathrm{el}}\left(R\right)=e^{\ln Z^{\mathrm{el}}\left(R\right)}=e^{-\beta F^{\mathrm{el}}\left(R\right)}$$

10 $$Z^{\mathrm{tot}}=\int Z^{\mathrm{el}}\left(R\right)dR=\int e^{-\beta F^{\mathrm{el}}\left(R\right)}dR$$

Equation 9 is a reformulation of the definition of the free energy in terms of the partition function, $F^{\mathrm{el}}\left(R\right)=-\frac{1}{\beta }\ln Z^{\mathrm{el}}\left(R\right)$ . With this, eq 6 looks like the usual equation for a Boltzmann factor, but with the electronic Helmholtz free energy at given orientations, *F* ^el^(**R**) instead of the total energy. Inserting eq 6 with eqs 9 and 10 into eq 8, one obtains

11 $$\langle B^{\mathrm{ind}}\rangle^{\mathrm{tot}}=\frac{\int e^{-\beta F^{\mathrm{el}}\left(R\right)}\langle B^{\mathrm{ind}}\rangle^{\mathrm{el}}\left(R\right)dR}{\int e^{-\beta F^{\mathrm{el}}\left(R\right)}dR}$$

In a commonly made approximation, one can assume that the electronic free energy *F* ^el^(**R**) is **R**-independent, which means all orientations are equally likely, and that ⟨**B** ^ind^⟩^el^(**R**) = −**σ** ^T^ **B**_0_. This leads to the well-known result that the chemical shift in solution is equal to one-third of the trace of the chemical shielding tensor **σ**. Note that the order of averaging implied by eq 11 (first electronic, then orientational) is exact, and does not depend on a separation of time scales (which would be problematic, because rotations and electronic relaxation can happen on similar time scales).

### 2.2 Infinite-Order Induced Field

Denoting the homogeneous external magnetic field as *B⃗*_0_, the total magnetic field felt by a magnetic nucleus is given by *B⃗* = *B⃗*_0_ + ⟨*B⃗* ^ind^⟩^tot^. The rotation matrix **R** describing the relation between the laboratory frame and the molecule-fixed frame can be parametrized with three Euler angles:^24^ θ and ϕ, the polar and azimuthal angles of *z* with respect to *Z*, and χ, which describes the rotation of the molecule-fixed frame around its own *z* axis. This Euler angle convention is also called the “extrinsic ZYZ” convention, because the molecule-fixed frame can be obtained by starting with the laboratory frame, then performing a rotation of all basis vectors around Z by angle χ, then performing a rotation around Y by angle θ, and finally performing a rotation around Z by angle ϕ. The rotation can be expressed in terms of the orthogonal rotation matrix or “direction cosine matrix”:^24^

12 $$R=R^{\left(Z,\phi \right)}R^{\left(Y,\theta \right)}R^{\left(Z,\chi \right)}=\left(\begin{array}{ccc} c\phi & -s\phi & 0\\ s\phi & c\phi & 0\\ 0 & 0 & 1 \end{array}\right)\left(\begin{array}{ccc} c\theta & 0 & s\theta \\ 0 & 1 & 0\\ -s\theta & 0 & c\theta \end{array}\right)\left(\begin{array}{ccc} c\chi & -s\chi & 0\\ s\chi & c\chi & 0\\ 0 & 0 & 1 \end{array}\right)=\left(\begin{array}{ccc} c\phi c\theta c\chi -s\phi s\chi & -c\phi c\theta s\chi -s\phi c\chi & c\phi s\theta \\ s\phi c\theta c\chi +c\phi s\chi & -s\phi c\theta s\chi +c\phi c\chi & s\phi s\theta \\ -s\theta c\chi & s\theta s\chi & c\theta \end{array}\right)$$

Here, “c” stands for cosine and “s” for sine. Any physical vector *v⃗* can be expressed in terms of either of the two frames, i.e.,

13 $$\mathrm{v⃗}=\sum_{I=X,Y,Z}v_{I}^{\mathrm{lab}}{\mathrm{e⃗}}_{I}=\sum_{i=x,y,z}v_{i}^{\mathrm{mol}}{\mathrm{e⃗}}_{i}=\sum_{i=x,y,z}v_{i}^{\mathrm{mol}}\sum_{I=X,Y,Z}{\mathrm{e⃗}}_{I}R_{\mathrm{Ii}}$$

This shows that the triples of vector components with respect to these two bases are related through **v** ^lab^ = **Rv** ^mol^. Note that for clarity, we choose a different notation for physical vectors, denoted by *v⃗*, and their representation in a basis, denoted by the triple of numbers **v**.

The average of a function *f*(**R**) = *f*(θ, ϕ, χ) over all possible orientations is given by

14 $$\langle f\rangle^{\mathrm{rot}}=\frac{\int f\left(R\right)dR}{\int dR}$$

where integration over orientations is defined as

15 $$\int dR=\int_{0}^{2\pi }d\chi \int_{0}^{2\pi }d\phi \int_{0}^{\pi }\sin\theta d\theta$$

The integration in the denominator can be performed analytically, such that eq 14 becomes

16 $$\langle f\rangle^{\mathrm{rot}}=\frac{1}{8\pi^{2}}\int_{0}^{2\pi }d\chi \int_{0}^{2\pi }d\phi \int_{0}^{\pi }\sin\left(\theta \right)d\theta f\left(\theta ,\phi ,\chi \right)$$

When averaging components of vectors or tensors (quantities that depend on the chosen frame) over different orientations, it is important that the *same frame* is used for all molecular orientations. The molecule-fixed frame is different for each molecular orientation. In the following, we will therefore average over components of the induced magnetic field expressed in the laboratory frame, not the molecule-fixed frame.

Without loss of generality, one can assume that the laboratory frame is chosen such that **B**_0_^lab^ = *B*_0_ (0, 0, 1)^T^, i.e., *B⃗*_0_ = *B*_0_ *e⃗*_*Z*_. Then the external field in the molecule-fixed frame (for a given orientation parametrized by Euler angles) is

17 $$B_{0}^{\mathrm{mol}}\left(\chi ,\theta \right)=R^{T}B_{0}^{\mathrm{lab}}=B_{0}\left(\begin{array}{c} -\sin\theta \cos\chi \\ \sin\theta \sin\chi \\ \cos\theta \end{array}\right)$$

One can see that this is *independent* of the third Euler angle ϕ. Using **B**_0_^mol^(χ, θ), one can calculate the electronically averaged induced field ⟨**B** ^ind^⟩^mol^(χ, θ) exactly (up to infinite order in the external field), i.e.,

$$\langle B^{\mathrm{ind}}\rangle^{\mathrm{mol}}\left(\chi ,\theta \right)=-\frac{1}{Z^{\mathrm{el}}\left(R\right)}\;\mathrm{tr}\left(\frac{\partial H}{\partial M^{\mathrm{mol}}}e^{-\beta H\left(R\right)}\right)=-\frac{1}{Z^{\mathrm{el}}\left(R\right)}\underset{i}{\sum }\langle i\left(R\right)|\frac{\partial H}{\partial M^{\mathrm{mol}}}|i\left(R\right)\rangle e^{-\beta E\left(i,R\right)}$$ 18

Here, **M** ^mol^ is the magnetic moment vector for the nucleus of interest, which, like the external field **B**_0_^mol^, is a parameter in the Hamiltonian *H*(**R**). Any observable expressed in the molecule-fixed frame will be independent from the Euler angle ϕ. This is in particular true for the electronic Helmholtz free energy *F*(**R**) = *F*(χ, θ), which is identical in the laboratory frame and the molecule-fixed frame since it is a scalar. Transforming the average induced field vector back to the laboratory frame gives the ⟨**B** ^ind^⟩^el^(**R**) occurring in eq 11:

19 $$\langle B^{\mathrm{ind}}\rangle^{\mathrm{lab}}\left(\chi ,\theta ,\phi \right)=R\langle B^{\mathrm{ind}}\rangle^{\mathrm{mol}}\left(\chi ,\theta \right)=\left(\begin{array}{c} \cos\phi \left(\cdot \cdot \cdot \right)+\sin\phi \left(\cdot \cdot \cdot \right)\\ \cos\phi \left(\cdot \cdot \cdot \right)+\sin\phi \left(\cdot \cdot \cdot \right)\\ 1\left(\cdot \cdot \cdot \right) \end{array}\right)$$

where the terms indicated by (···) are *independent* of ϕ. When performing the rotational average according to eq 11, the integration over ϕ can be done analytically for each of the three components and gives

20 $$\int_{0}^{2\pi }\cos\phi d\phi =\int_{0}^{2\pi }\sin\phi d\phi =0$$

21 $$\int_{0}^{2\pi }1d\phi =2\pi$$

This means that the *x* and *y* components of the induced field in the laboratory frame vanish after orientational averaging. Intuitively, one can recognize that after a 180° rotation of the molecule around the external magnetic field axis (change of the Euler angle ϕ by an angle of π), one has cos­(ϕ + π) = −cos­(ϕ) and sin­(ϕ + π) = −sin­(ϕ), which means that the *x* and *y* components of the induced magnetic field in the laboratory frame change sign. Furthermore, these two orientations have equal likelihood, since the external field in the molecule-fixed frame is the same. When averaging, the *x* and *y* components for each pair of orientations related by a 180° rotation exactly cancel each other. For the *z* component, from eq 11 we obtain

22 $$B^{\mathrm{ind}}=\langle \langle B_{z}^{\mathrm{ind}}\rangle^{\mathrm{lab}}\rangle^{\mathrm{rot}}=\frac{N}{D}$$

where, in order to keep the expressions short, we provide separate equations for the numerator *N* and the denominator *D* (with the common factor 2π from the analytical integration over ϕ already canceled):

23 $$N=\int_{0}^{2\pi }d\chi \int_{0}^{\pi }\sin\theta d\theta e^{-\beta F^{\mathrm{el}}\left(\chi ,\theta \right)}\langle B_{z}^{\mathrm{ind}}\rangle^{\mathrm{lab}}\left(\chi ,\theta \right)$$

24 $$\langle B_{z}^{\mathrm{ind}}\rangle^{\mathrm{lab}}\left(\chi ,\theta \right)=-\sin\theta \cos\chi \langle B_{x}^{\mathrm{ind}}\rangle^{\mathrm{mol}}\left(\chi ,\theta \right)+\sin\theta \sin\chi \langle B_{y}^{\mathrm{ind}}\rangle^{\mathrm{mol}}\left(\chi ,\theta \right)+\cos\theta \langle B_{z}^{\mathrm{ind}}\rangle^{\mathrm{mol}}\left(\chi ,\theta \right)$$

25 $$D=\int_{0}^{2\pi }d\chi \int_{0}^{\pi }\sin\theta d\theta e^{-\beta F^{\mathrm{el}}\left(\chi ,\theta \right)}$$

Equation 22 is valid to infinite order in the external field, i.e., for arbitrary field strengths.

### 2.3 Expansion of the Induced Field up to Third Order in the External Field

Starting from eq 22, we can expand the induced field in powers of the external field strength *B*_0_. In order to find the coefficients of this series expansion, one needs to compute the derivatives of the induced field, which are given by

26 $$\frac{\partial B^{\mathrm{ind}}}{\partial B_{0}}=\frac{\frac{\partial N}{\partial B_{0}}}{D}-\frac{N\frac{\partial D}{\partial B_{0}}}{D^{2}}$$

27 $$\frac{\partial^{2}B^{\mathrm{ind}}}{\partial B_{0}^{2}}=\frac{\frac{\partial^{2}N}{\partial B_{0}^{2}}}{D}-2\frac{\frac{\partial N}{\partial B_{0}}\frac{\partial D}{\partial B_{0}}}{D^{2}}-\frac{N\frac{\partial^{2}D}{\partial B_{0}^{2}}}{D^{2}}+2\frac{N{\left(\frac{\partial D}{\partial B_{0}}\right)}^{2}}{D^{3}}$$

28 $$\frac{\partial^{3}B^{\mathrm{ind}}}{\partial B_{0}^{3}}=\frac{\frac{\partial^{3}N}{\partial B_{0}^{3}}}{D}-3\frac{\frac{\partial^{2}N}{\partial B_{0}^{2}}\frac{\partial D}{\partial B_{0}}}{D^{2}}-3\frac{\frac{\partial N}{\partial B_{0}}\frac{\partial^{2}D}{\partial B_{0}^{2}}}{D^{2}}+6\frac{\frac{\partial N}{\partial B_{0}}{\left(\frac{\partial D}{\partial B_{0}}\right)}^{2}}{D^{3}}-\frac{N\frac{\partial^{3}D}{\partial B_{0}^{3}}}{D^{2}}+6\frac{N\frac{\partial^{2}D}{\partial B_{0}^{2}}\frac{\partial D}{\partial B_{0}}}{D^{3}}-6\frac{N{\left(\frac{\partial D}{\partial B_{0}}\right)}^{2}\frac{\partial D}{\partial B_{0}}}{D^{4}}$$

Next, we need to compute the derivatives of the numerator *N* and denominator *D*. In order to do so, it is convenient to define the integrands occurring in the numerator and denominator as

29 $$I_{N}=e^{-\beta F}\langle B_{z}^{\mathrm{ind}}\rangle^{\mathrm{lab}}=e^{-\beta F}\sum_{i}\langle B_{i}^{\mathrm{ind}}\rangle^{\mathrm{mol}}R_{\mathrm{Zi}}$$

30 $$I_{D}=e^{-\beta F}$$

In this section, we simply write *F* for *F* ^el^(χ, θ) and keep in mind that it is orientation-dependent (which is also the case for derivatives of *F*). We can express the electronically Boltzmann-averaged induced magnetic field (eq 18) as a derivative of the free energy:

31 $$\langle B_{i}^{\mathrm{ind}}\rangle^{\mathrm{mol}}=-\frac{\partial F}{\partial M^{\mathrm{mol},i}}=-F_{M^{i}}$$

Here, we introduced a simplified notation where the variables with respect to which the derivative is taken are written as subscripts. For simplicity, we drop the label “mol” since all vectors in the remainder of this section will be in the molecule-fixed frame. With this notation, the integrand in the numerator can be written as

32 $$I_{N}=-e^{-\beta F}\sum_{i}F_{M^{i}}R_{\mathrm{Zi}}$$

Both *F* and *F*_*M* ^*i*^_ are functions of the external magnetic field vector in the molecule-fixed frame, which has the components *B*_0_^mol,*i*^ = *B*_0_ *R*_*Zi*_. Therefore, for any quantity *f* that is a function of **B**_0_^mol^ (e.g., the free energy and its derivatives), we can use the chain rule to write

33 $$\frac{\partial f}{\partial B_{0}}=\sum_{i}f_{B_{0}^{i}}\frac{\partial B_{0}^{\mathrm{mol},i}}{\partial B_{0}}=\sum_{i}f_{B_{0}^{i}}R_{\mathrm{Zi}}$$

Using this result, the first derivative of *I*_D_ is given by

34 $$\frac{\partial I_{D}}{\partial B_{0}}=-\beta e^{-\beta F}\sum_{i}F_{B_{0}^{i}}R_{\mathrm{Zi}}$$

Comparing this with eq 32 for *I*_N_, we see that we can express both in terms of a single quantity *I*_**μ**_,

35 $$I_{N}=I_{M}$$

36 $$\frac{\partial I_{D}}{\partial B_{0}}=\beta I_{B_{0}}$$

Here,

37 $$I_{\mu }=-e^{-\beta F}\sum_{i}F_{\mu^{i}}R_{\mathrm{Zi}}$$

with **μ** being an arbitrary vector parameter (either **M** ^mol^ or **B**_0_^mol^ in our case). This allows us to derive the higher derivatives with respect to *B*_0_ only once, instead of doing this separately for the two integrands. These derivatives are given by

38 $$\frac{\partial I_{\mu }}{\partial B_{0}}=e^{-\beta F}\sum_{\mathrm{ij}}\left(\beta F_{\mu^{i}}F_{B_{0}^{j}}-F_{\mu^{i}B_{0}^{j}}\right)R_{\mathrm{Zi}}R_{\mathrm{Zj}}$$

39 $$\frac{\partial^{2}I_{\mu }}{\partial B_{0}^{2}}=e^{-\beta F}\sum_{\mathrm{ijk}}\left[-\beta^{2}F_{\mu^{i}}F_{B_{0}^{j}}F_{B_{0}^{k}}+\beta \left(2F_{\mu^{i}B_{0}^{j}}F_{B_{0}^{k}}+F_{\mu^{i}}F_{B_{0}^{j}B_{0}^{k}}\right)-F_{\mu^{i}B_{0}^{j}B_{0}^{k}}\right]R_{\mathrm{Zi}}R_{\mathrm{Zj}}R_{\mathrm{Zk}}$$

40 $$\frac{\partial^{3}I_{\mu }}{\partial B_{0}^{3}}=e^{-\beta F}\sum_{\mathrm{ijkl}}\left[\beta^{3}F_{\mu^{i}}F_{B_{0}^{j}}F_{B_{0}^{k}}F_{B_{0}^{l}}-3\beta^{2}\left(F_{\mu^{i}B_{0}^{j}}F_{B_{0}^{k}}F_{B_{0}^{l}}+F_{\mu^{i}}F_{B_{0}^{j}B_{0}^{k}}F_{B_{0}^{l}}\right)+\beta \left(3F_{\mu^{i}B_{0}^{j}B_{0}^{k}}F_{B_{0}^{l}}+F_{\mu^{i}}F_{B_{0}^{j}B_{0}^{k}B_{0}^{l}}\right)+3\beta F_{\mu^{i}B_{0}^{j}}F_{B_{0}^{k}B_{0}^{l}}-F_{\mu^{i}B_{0}^{j}B_{0}^{k}B_{0}^{l}}\right]R_{\mathrm{Zi}}R_{\mathrm{Zj}}R_{\mathrm{Zk}}R_{\mathrm{Zl}}$$

For a function *f*(χ, θ), integration over the two Euler angles can be expressed as (according to eq 16)­

41 $$\int_{0}^{2\pi }d\chi \int_{0}^{\pi }\sin\theta d\theta f=4\pi \langle f\rangle^{\mathrm{rot}}$$

For the reference field strength *B*_0_ = 0, the free energy and its derivatives become independent of the Euler angles, and the only part of the integrands that is dependent on the orientation is the product of the *R*_*Zi*_ factors. There exist simple analytical equations for rotational averages of such products,^24^ from which we obtain

42 $$\int_{0}^{2\pi }d\chi \int_{0}^{\pi }\sin\theta d\theta R_{\mathrm{Zi}}=0$$

43 $$\int_{0}^{2\pi }d\chi \int_{0}^{\pi }\sin\theta d\theta R_{\mathrm{Zi}}R_{\mathrm{Zj}}=\frac{4\pi }{3}\delta_{\mathrm{ij}}$$

44 $$\int_{0}^{2\pi }d\chi \int_{0}^{\pi }\sin\theta d\theta R_{\mathrm{Zi}}R_{\mathrm{Zj}}R_{\mathrm{Zk}}=0$$

45 $$\int_{0}^{2\pi }d\chi \int_{0}^{\pi }\sin\theta d\theta R_{\mathrm{Zi}}R_{\mathrm{Zj}}R_{\mathrm{Zk}}R_{\mathrm{Zl}}=\frac{4\pi }{15}\left(\delta_{\mathrm{ij}}\delta_{\mathrm{kl}}+\delta_{\mathrm{ik}}\delta_{\mathrm{jl}}+\delta_{\mathrm{il}}\delta_{\mathrm{jk}}\right)$$

Using these equations, the values of the numerator and the denominator and their derivatives at the reference field strength *B*_0_ = 0 are given by

46 $$D^{\left(0\right)}=4\pi e^{-\beta F^{\left(0\right)}}$$

47 $${\left(\frac{\partial D}{\partial B_{0}}\right)}^{\left(0\right)}=0$$

48 $${\left(\frac{\partial^{2}D}{\partial B_{0}^{2}}\right)}^{\left(0\right)}=-\beta 4\pi e^{-\beta F^{\left(0\right)}}\frac{1}{3}\sum_{i}F_{B_{0}^{i}B_{0}^{i}}^{\left(0\right)}$$

49 $${\left(\frac{\partial^{3}D}{\partial B_{0}^{3}}\right)}^{\left(0\right)}=0$$

and

50 $$N^{\left(0\right)}=0$$

51 $${\left(\frac{\partial N}{\partial B_{0}}\right)}^{\left(0\right)}=-4\pi e^{-\beta F^{\left(0\right)}}\frac{1}{3}\sum_{i}F_{M^{i}B_{0}^{i}}^{\left(0\right)}$$

52 $${\left(\frac{\partial^{2}N}{\partial B_{0}^{2}}\right)}^{\left(0\right)}=0$$

53 $${\left(\frac{\partial^{3}N}{\partial B_{0}^{3}}\right)}^{\left(0\right)}=4\pi e^{-\beta F^{\left(0\right)}}\sum_{\mathrm{ij}}\left[\frac{1}{5}\beta F_{M^{i}B_{0}^{i}}^{\left(0\right)}F_{B_{0}^{j}B_{0}^{j}}^{\left(0\right)}+\frac{2}{5}\beta F_{M^{i}B_{0}^{j}}^{\left(0\right)}F_{B_{0}^{i}B_{0}^{j}}^{\left(0\right)}-\frac{1}{5}F_{M^{i}B_{0}^{i}B_{0}^{j}B_{0}^{j}}^{\left(0\right)}\right]$$

Identifying the susceptibility tensor **χ** and chemical shielding tensor **σ** with second derivatives of the free energy, as well as the **τ** tensor with a fourth derivative of the free energy,

54 $$F_{M^{i}B_{0}^{j}}^{\left(0\right)}=\sigma_{\mathrm{ji}}$$

55 $$F_{B_{0}^{i}B_{0}^{j}}^{\left(0\right)}=-\frac{1}{\mu_{0}}\chi_{\mathrm{ij}}$$

56 $$F_{M^{i}B_{0}^{j}B_{0}^{k}B_{0}^{l}}^{\left(0\right)}=3!\tau_{\mathrm{jkli}}$$

one obtains

57 $$B^{\mathrm{ind}\left(0\right)}=0$$

58 $${\left(\frac{\partial B^{\mathrm{ind}}}{\partial B_{0}}\right)}^{\left(0\right)}=\frac{{\left(\frac{\partial N}{\partial B_{0}}\right)}^{\left(0\right)}}{D^{\left(0\right)}}=-\frac{1}{3}\sum_{i}\sigma_{\mathrm{ii}}=-\frac{1}{3}\sigma^{\left(2:1\right)}$$

59 $${\left(\frac{\partial^{2}B^{\mathrm{ind}}}{\partial B_{0}^{2}}\right)}^{\left(0\right)}=0$$

60 $${\left(\frac{\partial^{3}B^{\mathrm{ind}}}{\partial B_{0}^{3}}\right)}^{\left(0\right)}=\frac{{\left(\frac{\partial^{3}N}{\partial B_{0}^{3}}\right)}^{\left(0\right)}}{D^{\left(0\right)}}-3\frac{{\left(\frac{\partial N}{\partial B_{0}}\right)}^{\left(0\right)}{\left(\frac{\partial^{2}D}{\partial B_{0}^{2}}\right)}^{\left(0\right)}}{{\left(D^{\left(0\right)}\right)}^{2}}$$

61 $$=\frac{2}{15}\frac{\beta }{\mu_{0}}\sigma^{\left(2:1\right)}\chi^{\left(2:1\right)}-\frac{2}{5}\frac{\beta }{\mu_{0}}{\left(\sigma \chi \right)}^{\left(2:1\right)}-\frac{3!}{5}\tau^{\left(4:2\right)}$$

Here, (*n*:*l*) denotes the *l*-fold trace (i.e., contraction of *l* pairs of indices) of an *n*th order tensor.^25^ The (2:1) trace is equal to the usual trace of a matrix, e.g., **σ** ^(2:1)^ = ∑_*i*_σ_*ii*_, and **τ** ^(4:2)^ = ∑_*ij*_τ_*iijj*_. Up to third order in the field strength *B*_0_, we then get

62 $$B^{\mathrm{ind}}\approx {\left(\frac{\partial B^{\mathrm{ind}}}{\partial B_{0}}\right)}^{\left(0\right)}B_{0}+\frac{1}{3!}{\left(\frac{\partial^{3}B^{\mathrm{ind}}}{\partial B_{0}^{3}}\right)}^{\left(0\right)}B_{0}^{3}=-\frac{1}{3}\sigma^{\left(2:1\right)}B_{0}+\left[\frac{1}{45}\frac{\beta }{\mu_{0}}\sigma^{\left(2:1\right)}\chi^{\left(2:1\right)}-\frac{1}{15}\frac{\beta }{\mu_{0}}{\left(\sigma \chi \right)}^{\left(2:1\right)}-\tau \right]B_{0}^{3}$$

where we introduced^3,16^

63 $$\tau =\frac{1}{5}\tau^{\left(4:2\right)}$$

The constant τ was first discussed by Ramsey for diamagnetic molecules.^10^

We can use eq 62 to write the total field experienced by the nucleus as

64 $$B=B_{0}+B^{\mathrm{ind}}=\left(1-\sigma -\sigma^{\left(2\right)}B_{0}^{2}\right)B_{0}+O\left(B_{0}^{4}\right)$$

Here, σ is the normal field-independent isotropic shielding constant and σ^(2)^ incorporates the effect of both indirect and direct field dependence. The NMR shift is defined as (relative to a reference compound “ref”)

65 $$\delta =\frac{\nu -\nu_{\mathrm{ref}}}{\nu_{\mathrm{ref}}}=\frac{B-B_{\mathrm{ref}}}{B_{\mathrm{ref}}}$$

which can be written up to second order in *B*_0_ as

66 $$\delta =\frac{\sigma_{\mathrm{ref}}-\sigma }{1-\sigma_{\mathrm{ref}}}+\frac{(1-\sigma )\sigma_{\mathrm{ref}}^{\left(2\right)}-\left(1-\sigma_{\mathrm{ref}}\right)\sigma^{\left(2\right)}}{{\left(1-\sigma_{\mathrm{ref}}\right)}^{2}}B_{0}^{2}+O\left(B_{0}^{3}\right)$$

Using the approximation 1 – σ_ref_ ≈ 1 – σ ≈ 1, this simplifies to

67 $$\delta \approx \sigma_{\mathrm{ref}}-\sigma +\left(\sigma_{\mathrm{ref}}^{\left(2\right)}-\sigma^{\left(2\right)}\right)B_{0}^{2}$$

If we are just interested in the paramagnetic part of the shift, and assume that the paramagnetic contribution to the induced field for the reference compound is zero, we end up at the following estimate for the shift:

68 $$\delta \approx \frac{B^{\mathrm{ind}}}{B_{0}}\approx -\sigma -\sigma^{\left(2\right)}B_{0}^{2}=-\frac{1}{3}\sigma^{\left(2:1\right)}+\left[\frac{1}{45}\frac{\beta }{\mu_{0}}\sigma^{\left(2:1\right)}\chi^{\left(2:1\right)}-\frac{1}{15}\frac{\beta }{\mu_{0}}{\left(\sigma \chi \right)}^{\left(2:1\right)}-\tau \right]B_{0}^{2}$$

The first term is the familiar field-independent shift. The first two terms in square brackets correspond to the well-known correction from partial self-orientation of the molecules in the magnetic field,^6,7^ i.e., the “indirect”^3^ contribution to the field dependence. And the final term in square brackets is the “direct”^3^ contribution to field dependence.

A note is in order on the physical interpretation of the **σ** and **τ** tensors in diamagnetic versus paramagnetic molecules. For diamagnetic molecules, the wavefunction has to change in response to the external magnetic field. This changed wavefunction creates an additional magnetic field (“induced field”). In the absence of the external field, the ground state wavefunction does not create a magnetic field, since there are no orbital currents and no unpaired spins. In contrast, if we, e.g., treat a paramagnetic spin 1/2 system in the spin Hamiltonian approximation, then the two states are completely independent of the field strength and are equal to the states at zero field. The difference to the diamagnetic case is that already those states in zero fields create a *permanent* magnetic field. It is just that at zero field, both states (creating opposite magnetic fields) have exactly equal populations, such that the created magnetic fields average to zero due to electronic fluctuations. If we apply an external field, we create a population difference (because of the energy splitting), meaning that the permanent magnetic fields of the two states will not exactly cancel, and there will be a net “induced field”. At large enough fields, saturation effects will appear, until eventually (at room temperature for fields that are much larger than what can routinely be produced in the laboratory) only the lower-energy state is populated. For example, at room temperature (298 K) and 28.2 T (the magnetic induction inside a 1.2 GHz spectrometer), the lower Zeeman level of a spin 1/2 system with isotropic *g*-value (*g* ≈ 2) will have a population of around 53%, i.e., only slightly larger than the population of the upper level. In contrast, at 1000 T, the population of the lower level would reach around 99%. At this point, the “induced field” is constant, and no longer linear in the external field. Similar to how the **σ** tensor is due to population differences in the linear regime, these saturation effects are the major origin of the **τ** tensor for paramagnetic molecules. It should be stressed that the formalism developed herein is a generalization of the diamagnetic case (since the Helmholtz free energy becomes equal to the ground state energy for diamagnetic molecules at reasonable temperatures), so the proper response of the wavefunction is also contained in the general equations for the fourth derivative of the Helmholtz free energy. It is just that for paramagnetic molecules, these diamagnetic effects are not dominant, and are neglected in the computational results of this work (see below).

## 3 Implementation

### 3.1 Finite-Field and Second-Order Approach for the Calculation of Field-Dependent Shifts

Given the theory developed so far, there are two ways to compute field-dependent NMR shifts, both with advantages and disadvantages. The first option is the computation of finite-field shifts by doing the rotational averaging numerically. To obtain *N* and *D*, the two integrals given by eqs 23 and 25 can be solved numerically by introducing a grid (χ_*i*_, θ_*i*_) on the unit sphere and evaluating ⟨**B** ^ind^⟩^mol^(χ_*i*_, θ_*i*_) for each grid point. The double integration over χ and θ can then be replaced by a summation over grid points. We call this the finite-field approach. In this work, we use Lebedev grids^26^ to perform the numerical integration. The second option consists in computing the **σ**, **χ**, and **τ** tensors analytically as derivatives of the Helmholtz free energy and using eq 68 to obtain the shift. For **σ** and **χ**, which are second derivatives of the free energy, the analytical sum-over-states equations (Van Vleck, Van den Heuvel–Soncini) are well-known. In order to calculate the **τ** tensor, we generalized our previous rederivation of the Van den Heuvel–Soncini equation (Appendix of Reference 27) to obtain sum-over-states expressions for analytical third and fourth derivatives of the free energy. This highly nontrivial derivation, which is quite technical, is presented in the Appendix. In order to evaluate the fourth derivative of the free energy, the same ingredients as for the second derivatives are needed: Eigenstates and energies of the field-free Hamiltonian, and derivatives of the Hamiltonian with respect to the parameters occurring in the derivative. The correctness of the analytical derivatives was checked by comparison with numerical derivatives.

The advantages and disadvantages of the two approaches are listed in Table 1. From that comparison, it becomes clear that the second-order approach is usually preferable except for the caveat that it will eventually break down for too strong fields/too low temperatures. The finite-field approach should only be used to check the validity of the second-order approach (see Computational Results below) and in cases where the second-order approach is known to fail.

**Table 1:** Comparison of the Advantages (+) and Disadvantages (−) of the Finite-Field and Second-Order Approaches

finite-field approach	second-order approach
+ valid for arbitrarily strong fields/arbitrarily low temperatures	– shifts limited to second order in *B*_0_
– average over orientations is approximate: numerical errors	+ average over orientations is exact/analytical
– harder to interpret	+ easier to interpret
– each field strength requires a separate calculation	+ the shift can be obtained at different field strengths from just one calculation

The analytical sum-over-states equations derived in the Appendix are applicable in the case that the states and energies of the molecule are calculated by exactly diagonalizing some Hamiltonian *H*(**R**) describing the system in the molecule-fixed frame (which does not necessarily need to be the exact *ab initio* electronic Hamiltonian). Recall that this Hamiltonian is orientation-dependent because **B**_0_^mol^(**R**) is orientation-dependent. Possible choices for this Hamiltonian are for example the *ab initio* quasidegenerate perturbation theory (QDPT) effective Hamiltonian based on CASSCF/NEVPT2 nonrelativistic/scalar-relativistic wavefunctions,^28^ the ligand field theory (LFT) effective Hamiltonian, or various kinds of spin Hamiltonians.^29^ For quantum-chemical wavefunction parametrizations that are not based on the exact diagonalization of some Hamiltonian, one would need to derive specific analytical formulae for the analytical derivatives of the free energy, which probably would involve the solution of wavefunction response equations instead of an explicit summation over field-free excited states.

### 3.2 Computational Model Used Herein

We illustrate the approaches developed herein using the NiSAL–HDPT complex^30,31^ as an example. We follow a computational strategy similar to the one recently established for the calculation of field-independent shifts of this complex.^22^ For simplicity, we neglect the diamagnetic contribution to shielding and focus on the paramagnetic Fermi contact (FC) and pseudocontact (PC) shifts. The division into these two contributions is not unambiguous. Within the spin Hamiltonian approximation, the former corresponds to the part caused by the isotropic hyperfine coupling constant, while the latter derives from the anisotropic part of the hyperfine coupling tensor **A**.

In the following equations, we use Hartree atomic units. We model the FC shifts within the spin Hamiltonian approximation. For the finite-field approach, for each orientation separately, we diagonalize

69 $$H_{\mathrm{spin}}=S^{T}\mathrm{DS}+\frac{1}{2}B_{0}^{T}\mathrm{gS}$$

The prefactor of 1/2 in the Zeeman term is the Bohr magneton μ_B_ = *e*ℏ/2*m*_e_ in atomic units. Note that we leave out hyperfine coupling when diagonalizing this Hamiltonian. In principle, hyperfine coupling leads to an additional contribution to the induced magnetic field (next to the one due to the external field). As a consequence of leaving out this contribution, indirect nuclear spin–spin coupling is not described here. Also note that **B**_0_ and **S**, as well as the spin Hamiltonian parameters, refer to the molecule-fixed frame. The field-dependent states, energies, and partition functions obtained after diagonalization of eq 69 are inserted into eq 18 to compute the electronically-averaged induced field in the molecule-fixed frame. In that equation,

70 $$\frac{\partial H}{\partial M^{\mathrm{mol}}}=\frac{A_{\mathrm{iso}}}{\gamma }S$$

where we only consider the isotropic hyperfine coupling constant.

We model the PC shifts using the LFT Hamiltonian and the point-dipole approximation (PDA).^27,32^ We employ parameters obtained from *ab initio* ligand field theory (AILFT).^33–36^ For the finite-field approach, we diagonalize the exact LFT Hamiltonian in the presence of the external field **B**_0_ for each orientation and compute the electronically-averaged induced field according to eq 18 by using the operator

71 $$\frac{\partial H}{\partial M^{\mathrm{mol}}}=-\frac{3\alpha^{2}}{r^{3}}{\left(\mathrm{r̂}⊗\mathrm{r̂}\right)}^{\mathrm{aniso}}M^{\mathrm{el}}$$

Here, **r̂** is the unit vector pointing from the paramagnetic center (in the NiSAL–HDPT complex: the location of the Ni nucleus) to the magnetic nucleus, *r* is the distance between paramagnetic center and nucleus, (**r̂** ⊗ **r̂**)^aniso^ denotes the anisotropic/traceless part of the dyadic tensor constructed from **r̂**, i.e., ${\left(\mathrm{r̂}⊗\mathrm{r̂}\right)}_{ij}^{\mathrm{aniso}}={\mathrm{r̂}}_{i}{\mathrm{r̂}}_{j}-\frac{1}{3}\delta_{ij}$ , and the electronic magnetic moment operator is given by

72 $$M^{\mathrm{el}}=-\frac{\partial H}{\partial B_{0}^{\mathrm{mol}}}=-\frac{1}{2}L-S$$

with **L** and **S** being the total orbital and spin angular momentum operators in the LFT model.

For the second-order approach, one needs to calculate **σ**, **χ**, and **τ** from the analytical sum-over-states equations using the eigenstates and energy eigenvalues of the field-free Hamiltonian. For the FC shifts, the field-free Hamiltonian is **S** ^T^ **DS** and for the PC shifts, it is the LFT Hamiltonian in the absence of the field. Furthermore, the Hamiltonian derivatives with respect to the nuclear magnetic moment and external field are needed. The latter is given by

73 $$M^{\mathrm{el}}=-\frac{\partial H}{\partial B_{0}^{\mathrm{mol}}}=-\frac{1}{2}\mathrm{gS}$$

in the spin Hamiltonian approximation (for FC shifts) and by eq 72 in the LFT model (for PC shifts). The structure of the models used herein (spin Hamiltonian approximation and PDA) leads to a computational simplification: For the FC shifts, both Hamiltonian derivatives are linear in the spin operators, see eqs 70 and 73. Therefore, we can evaluate the quantities ⟨⟨*S*_*i*_ *S*_*j*_⟩⟩ (the spin dyadic tensor) and ⟨⟨*S*_*i*_ *S*_*j*_ *S*_*k*_ *S*_*l*_⟩⟩ (which can be called the “spin tetradic” tensor) and from these obtain the three needed tensors. See the Appendix for the “response theory” notation used here. Note that our definition of the spin dyadic differs by a factor of −β from the spin dyadic ⟨*S*_*i*_ *S*_*j*_⟩ of Vaara and co-workers^37^ (also used by us previously^27,32^ ), which is equal to the **Z**-matrix introduced by Autschbach and co-workers:^38^

74 $$\left\langle \left\langle S_{i}S_{j}\right\rangle \right\rangle =-\beta \left\langle S_{i}S_{j}\right\rangle =-\beta Z_{\mathrm{ij}}$$

In this way, the entire temperature dependence is contained in the spin dyadic. Similarly, considering eq 71 used for describing PC shifts, which is linear in the electronic magnetic moment operator, we only need the quantities ⟨⟨*M*_*i*_^el^ *M*_*j*_^el^⟩⟩ (apart from a prefactor, the susceptibility tensor) and ⟨⟨*M*_*i*_^el^ *M*_*j*_^el^ *M*_*k*_^el^ *M*_*l*_^el^⟩⟩ (apart from a prefactor, the second hypersusceptibility tensor) to obtain the three needed tensors. These quantities [spin dyadic/tetradic and (hyper)­susceptibilities] are computationally simpler to evaluate than directly calculating the **σ** or **τ** tensors: since they only involve the components of a single vector operator, they are (super)­symmetric (invariant under all permutations of their indices).

We implemented the approaches derived herein in a new Julia package called ParaMag.jl. The LFT implementation uses a Slater determinant basis and the formalism of second quantization; see section S1 of the Supporting Information for more details.

## 4 Computational Results

We tested the developed computational approaches by comparing experimental and calculated NMR shifts of the NiSAL–HDPT complex at 400 MHz and 1.2 GHz proton Larmor frequency. SAL-HDPT is a pentadentate ligand formed from two equivalents of salicylaldehyde (SAL) and dipropylenetriamine (DPT; H indicates that there is an H atom bound to the central amine in addition to the two propylene groups). NiSAL–HDPT was among the first high-spin pentacoordinate complexes of nickel to be obtained.^30,31^ In Figure 2, we display the structure of NiSAL–HDPT together with the proton labels as well as the comparison of a part of the spectrum at low and high field. Details on how the spectra and the experimental chemical shift values were obtained can be found in Section S2 of the Supporting Information. Experimentally, we observed that the magnitudes of the chemical shift values become smaller when moving to higher field, with the field-dependent variation being mostly less than 1% of the absolute shift value. The largest differences between low-field and high-field chemical shift values could be observed for the most shielded and deshielded protons. The computational details for obtaining the LFT and spin Hamiltonian parameters using ORCA^39–41^ are described in Section S3 of the Supporting Information. The left side of Figure 3 shows the relative deviation of the finite-field chemical shifts in NiSAL–HDPT with respect to the shifts obtained with the largest Lebedev grid, i.e., |δ −δ^largest^|/|δ^largest^| at 400 MHz proton Larmor frequency and a temperature of 298 K. In total, we have implemented 32 Lebedev grids that have between 6 and 5810 grid points. Hence, the plot contains 31 data points. It can be observed that starting from the third grid, which has only 26 grid points, the relative deviation is reliably below 10^–8^. Further improvement with increasing the grid size is slow and erratic. For the largest shifts in NiSAL, which occur at around 400 ppm, a relative deviation of 10^–8^ corresponds to an error of around 4 × 10^–6^ ppm, which is negligible. Hence, accurate finite-field shifts can already be obtained with very moderate grid sizes. In Section S4 of the Supporting Information, we show that similar results are obtained when using REPULSION grids.^42^

**Figure 2:**
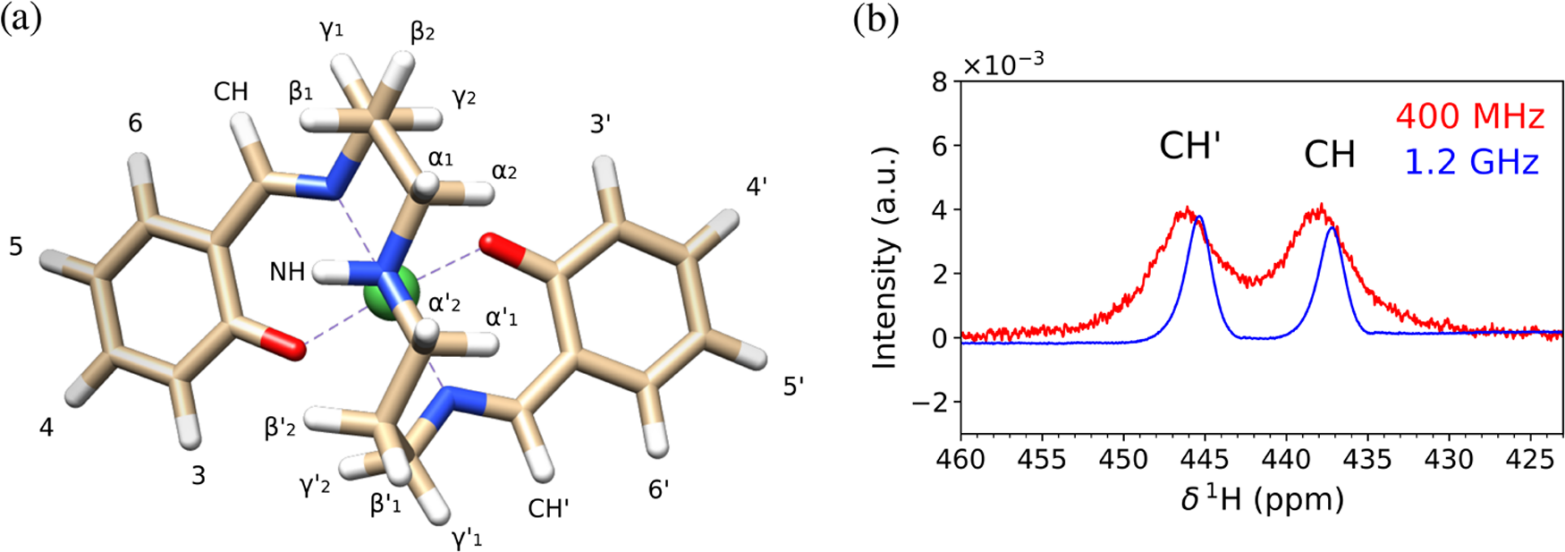
(a) Structure and proton labels of NiSAL–HDPT. (b) Part of the spectrum at low and high field. One can clearly see that the peak positions are field-dependent.

**Figure 3:**
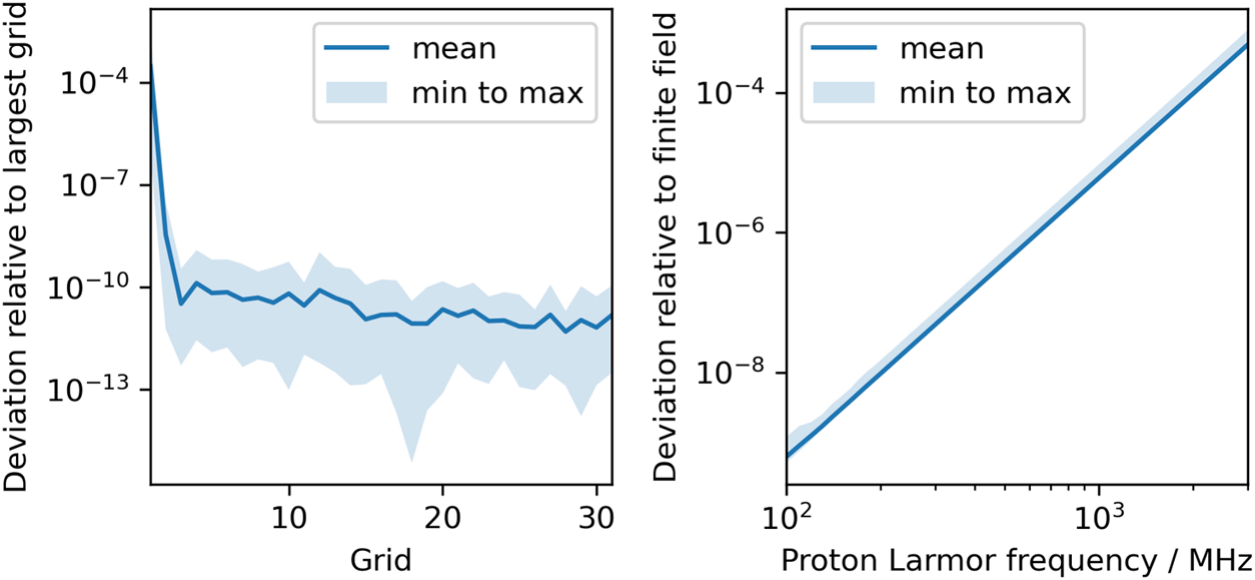
Left: Convergence of the finite-field approach with respect to the size of the Lebedev grid. Plotted is the *relative absolute deviation* with respect to the result obtained with the largest grid. The solid line is the mean over all protons, whereas the shaded area is the region between the smallest and largest value among all protons. Right: Relative absolute deviation of shifts obtained in the second-order approach from shifts obtained in the finite-field approach as a function of the field strength (measured in proton Larmor frequency). Below around 100 MHz, the deviation between the two approaches becomes smaller than the error introduced by the finite grid, which is why we do not show this region.

On the right side of Figure 3, we show the relative absolute deviation of the second-order shifts from the finite-field shifts, |δ^2nd^ – δ^finite^|/|δ^finite^|, as a function of the field strength (expressed as proton Larmor frequency). One can first observe that the deviation between the two approaches vanishes for small field strengths, which confirms the correctness of our derivation of the second-order approach and, in particular, of the sum-over-states equation for the **τ** tensor. Furthermore, one can see that the deviation has a linear appearance in the log–log plot, which means that it is a polynomial function of the proton Larmor frequency. One can observe that one order or magnitude change in the Larmor frequency (from 100 MHz to 1 GHz) corresponds to a four orders of magnitude increase in the relative absolute deviation of the second-order approach from the finite-field approach. This observation is easily explained: When expanding the shift in powers of *B*_0_, odd powers vanish exactly because of symmetry. The second-order approach describes the shifts correctly up to order *B*_0_^2^. Therefore, the leading order term in the difference of second-order and finite-field shifts is a *B*_0_^4^ term. One can see that at 1.2 GHz (corresponding to the largest commercially available field strength), the relative absolute deviation is still on the order of 10^–5^, which for the largest shifts in NiSAL of around 400 ppm corresponds to around 4 × 10^–3^ ppm. This deviation is in principle measurable in high-resolution NMR spectrometers but still much smaller than the second-order field-dependent shift, which has a size of around 1 ppm for the most shielded and deshielded protons in NiSAL–HDPT (see below) and is also much smaller than the width of the paramagnetically broadened peaks. This is evidence that the second-order approach, which has a couple of advantageous properties as summarized in Table 1, is sufficient at room temperature for the field strengths that can nowadays be routinely achieved. In Section S5 of the Supporting Information, we demonstrate that the validity of the second-order approach also extends to lower temperatures.

Next, we compare the experimental shift values with those calculated with the second-order approach. Some protons are excluded from the comparison because of large experimental uncertainties; see Section S2 of the Supporting Information. In Table 2, we list the experimental and calculated shifts at 400 MHz as well as the changes Δδ when moving from 400 MHz to 1.2 GHz. For the calculated shifts, we furthermore show the decomposition into FC and PC contributions. A visual representation of these numbers can be found in Figure 4. The left side of the figure shows the correlation between experimental shifts and those calculated with the second-order approach at 400 MHz. The agreement is very good and comparable to our previous work.^22^ Note, however, that we did not consider diamagnetic contributions to the shifts here, which is why the agreement is probably somewhat worse when focusing on the diamagnetic region only. On the absolute shift scale of several hundred ppm, the field-dependent contributions to the shifts at 400 MHz do not play a big role for the overall agreement with the experiment. Therefore, the right side of the same figure shows the correlation between experimental and calculated (second-order approach) *differences* between 1.2 GHz shifts and 400 MHz shifts. Again, the agreement is good, although not as excellent as the agreement of the absolute shifts. This could potentially originate from errors in extracting the experimental shifts, since the peaks are significantly broadened due to paramagnetic relaxation enhancement. These proof-of-principle results are the first evidence that our new approach for calculating field-dependent NMR shifts of paramagnetic molecules in solution is able to describe the phenomenon correctly.

**Table 2:** Experimental Shifts and Second-Order Calculated Shifts δ of NiSAL–HDPT at 400 MHz and Difference Δδ = δ­(1.2 GHz) – δ­(400 MHz) (All in ppm)^a^

			δ_calc_(400 MHz)			Δδ_calc_		
proton	δ_exp_(400 MHz)	Δδ_exp_	FC	PC	total	FC	PC	total
3′	–13.535	0.033	–17.424	–6.352	–23.776	0.047	0.012	0.059
4′	25.246	–0.053	27.306	–3.368	23.938	–0.073	0.006	–0.067
CH′	446.273	–0.986	422.938	26.187	449.125	–1.137	–0.049	–1.186
γ_2_	116.32	–0.111	64.435	57.62	122.055	–0.173	–0.106	–0.279
γ_1_	229.201	–0.321	192.422	35.674	228.096	–0.517	–0.066	–0.583
γ_2_′	110.991	–0.192	75.102	54.942	130.044	–0.202	–0.102	–0.304
γ_1_′	186.455	–0.292	168.368	36.254	204.622	–0.453	–0.068	–0.521
3	–13.996	0.016	–14.86	–5.66	–20.52	0.04	0.011	0.051
4	26.214	–0.055	28.241	–3.122	25.119	–0.076	0.006	–0.07
CH	438.033	–0.881	430.899	25.885	456.784	–1.159	–0.048	–1.207
α_2_	79.71	–0.231	95.651	–32.545	63.106	–0.257	0.061	–0.196
α_1_	286.931	–0.441	302.597	–19.992	282.605	–0.813	0.038	–0.775
α_2_′	258.472	–0.894	294.614	–17.234	277.38	–0.792	0.033	–0.759
β_2_′	–8.579	0.028	–12.82	–2.154	–14.974	0.035	0.005	0.04
β_1_′	–10.609	0.025	–14.644	8.481	–6.163	0.04	–0.015	0.025
NH	–333.074	0.824	–271.209	–84.399	–355.608	0.729	0.16	0.889

a Some protons with large experimental uncertainties are excluded from the comparison (see Section S2 of the Supporting Information)

**Figure 4:**
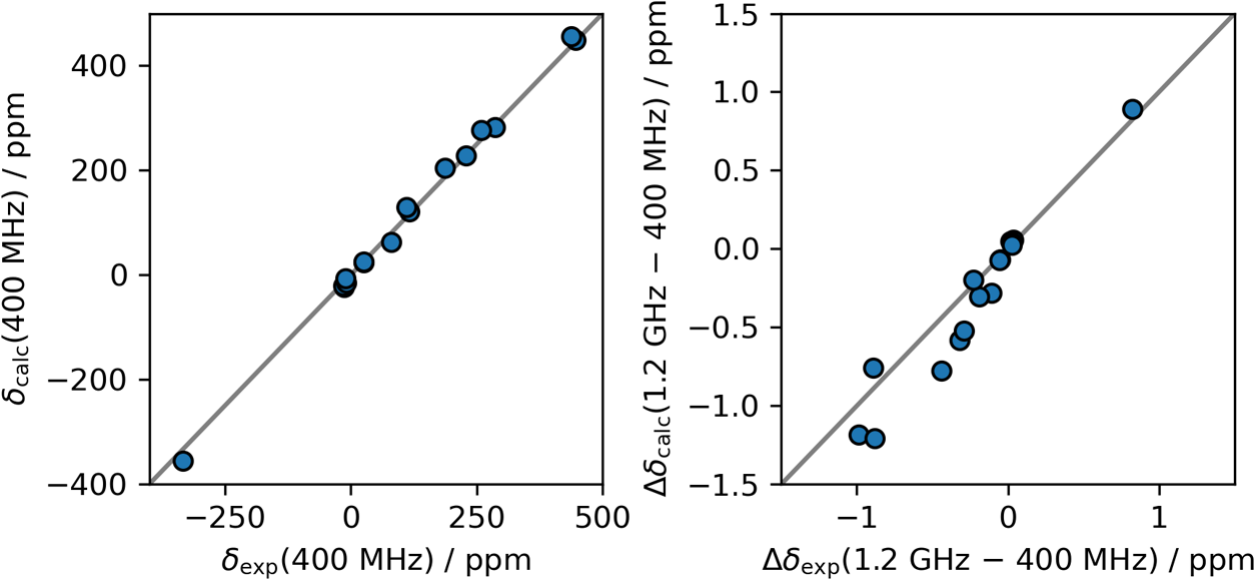
Left: Correlation plot of calculated versus experimental shifts of NiSAL–HDPT at 400 MHz proton Larmor frequency. Right: Correlation plot of calculated versus experimental differences between 1.2 GHz and 400 MHz shifts of NiSAL–HDPT. In both plots, the gray line corresponds to perfect correlation. The temperature is 298 K.

## 5 Conclusions

We derived working equations for calculating field-dependent NMR chemical shifts in paramagnetic molecules in solution, leading to two different computational approaches. The “finite-field approach” is valid for arbitrarily high field strengths and arbitrarily low temperatures, but has the disadvantage that it requires numerical integration to account for the orientational averaging in solution. Furthermore, we also developed a “second-order approach” that is only valid up to second order in the external magnetic flux density *B*_0_, i.e., in the weak field/high-temperature regime. This approach has the advantage that the orientational averaging can be performed analytically. Furthermore, at this level of theory, the field-dependent part of the shift separates cleanly into two additive contributions: An “indirect effect” that is due to the partial orientation of molecules with an anisotropic susceptibility tensor in solution and which takes exactly the form that is well-known in the literature, and a “direct effect” that originates from the nonlinear response of the system to the external field and depends on a generalization of the fourth-order **τ** tensor introduced by Ramsey, Vaara, and others for diamagnetic molecules. In order to calculate this **τ** tensor, we derived analytical sum-over-states equations for fourth derivatives of the electronic Helmholtz free energy, which generalize the Van Vleck and Van den Heuvel–Soncini equations that are examples for second derivatives of the Helmholtz free energy. We expect the new sum-over-states equations to have applications also outside the realm of NMR. We tested the newly developed approaches using the NiSAL–HDPT complex as an example, describing the paramagnetic shifts as the sum of Fermi contact shifts (modeled within the spin Hamiltonian approximation) and pseudocontact shifts (modeled within the ligand field theory approximation and employing the point-dipole approximation). It turned out that the finite-field approach converges very quickly with the size of the integration grid, and already a modest 26-point Lebedev grid is sufficient for accurate results. Furthermore, we could show that at room temperature and at the field strengths that can routinely be achieved, the second-order approach is sufficient for describing the field dependence of the shifts. Finally, we could demonstrate good agreement between the experimental and calculated field dependence of the shifts in the NiSAL–HDPT complex. In upcoming work, we will investigate the performance of the methods introduced herein in more detail for both transition metal and lanthanoid complexes in order to establish trends and develop an intuitive understanding of the field dependence of NMR shifts in paramagnetic molecules. Furthermore, we plan the implementation of the approaches developed herein beyond model Hamiltonians, e.g., at the level of the *ab initio* QDPT method.

## Supplementary Material



## Data Availability

The data underlying this study are openly available on Zenodo at 10.5281/zenodo.15334208. All calculations were performed with the ParaMag.jl Julia package, which can be obtained from https://github.com/LucasLang/ParaMag.jl and 10.5281/zenodo.15028574. A Snakemake^43^ workflow for reproducing the ParaMag.jl calculations and data analysis can be obtained from https://github.com/LucasLang/fielddepshifts_analysis and 10.5281/zenodo.15334203.

## Appendix: Analytical Derivatives of the Free Energy

In the following, we will make extensive use of Wilcox’s equation for the derivative of an exponential operator,^44^

75
$$\frac{\partial }{\partial \lambda }e^{-\beta H}=-\int_{0}^{\beta }e^{-\left(\beta -\omega \right)H}\frac{\partial H}{\partial \lambda }e^{-\omega H}d\omega$$

The electronic Helmholtz free energy is given by

76
$$F=-\frac{1}{\beta }\ln Z$$

where the canonical partition function is

77
$$Z=\mathrm{tr}\left(e^{-\beta H}\right)$$

or, in an eigenbasis of the Hamiltonian,

78
$$Z=\sum_{M\mu }e^{-\beta E_{M}}$$

Here, the index *M* runs over all eigenenergies of *H* and the index μ runs over all states having energy *E*_*M*_, i.e., it takes care of possible degeneracies. In the following, we will derive analytical expressions for derivatives of the free energy with respect to parameters like the external magnetic field or nuclear magnetic moments (average magnetic moment, susceptibility, shielding, and beyond) up to fourth order. We will assume for simplicity that all derivatives of the Hamiltonian beyond first derivatives vanish, as is, e.g., the case in the LFT Hamiltonian or the Dirac Hamiltonian (i.e., there are no “diamagnetic” terms). We will use the generic notation *λ* ^*i*^, *λ* ^*j*^, *λ* ^*k*^, *λ* ^*l*^ for the different parameters in the following.

### A.1 First Derivative of the Free Energy

Using the chain rule, one obtains from eq 76

79
$$\frac{\partial F}{\partial \lambda^{l}}=-\frac{1}{\beta }\frac{1}{Z}\frac{\partial Z}{\partial \lambda^{l}}$$

Using Wilcox’s equation, the derivative of the partition function is

80
$$\frac{\partial Z}{\partial \lambda^{l}}=-\beta \mathrm{tr}\left(\frac{\partial H}{\partial \lambda^{l}}e^{-\beta H}\right)$$

Inserting this expression gives

81
$$\frac{\partial F}{\partial \lambda^{l}}=\frac{1}{Z}\mathrm{tr}\left(\frac{\partial H}{\partial \lambda^{l}}e^{-\beta H}\right)$$

By inserting an eigenbasis of the Hamiltonian, this becomes

82
$$\frac{\partial F}{\partial \lambda^{l}}=\frac{1}{Z}\sum_{M\mu }\left\langle M\mu \left|\frac{\partial H}{\partial \lambda^{l}}\right|M\mu \right\rangle e^{-\beta E_{M}}$$

This is simply the Boltzmann average of the observable corresponding to the first derivative of the Hamiltonian.

### A.2 Second Derivative of the Free Energy

Starting from eq 81 and using the product rule, we obtain for the second derivative of the free energy

83
$$\frac{\partial^{2}F}{\partial \lambda^{k}\partial \lambda^{l}}=-\frac{1}{Z^{2}}\frac{\partial Z}{\partial \lambda^{k}}\mathrm{tr}\left(\frac{\partial H}{\partial \lambda^{l}}e^{-\beta H}\right)+\frac{1}{Z}\frac{\partial }{\partial \lambda^{k}}\mathrm{tr}\left(\frac{\partial H}{\partial \lambda^{l}}e^{-\beta H}\right)$$

Here and in the following, we use eq 79 to express the derivative of the partition function in terms of the first derivative of the free energy

84
$$\frac{\partial Z}{\partial \lambda^{k}}=-\beta Z\frac{\partial F}{\partial \lambda^{k}}$$

For the second term in eq 83, we apply Wilcox’s equation to arrive at the result

85
$$\frac{\partial^{2}F}{\partial \lambda^{k}\partial \lambda^{l}}=\beta \frac{\partial F}{\partial \lambda^{k}}\frac{\partial F}{\partial \lambda^{l}}-\frac{1}{Z}\int_{0}^{\beta }\mathrm{tr}\left(\frac{\partial H}{\partial \lambda^{l}}e^{-\left(\beta -\omega_{1}\right)H}\frac{\partial H}{\partial \lambda^{k}}e^{-\omega_{1}H}\right)d\omega_{1}$$

When inserting an eigenbasis of the Hamiltonian, one obtains for the second term

86
$$-\frac{1}{Z}\int_{0}^{\beta }\mathrm{tr}\left(\frac{\partial H}{\partial \lambda^{l}}e^{-\left(\beta -\omega_{1}\right)H}\frac{\partial H}{\partial \lambda^{k}}e^{-\omega_{1}H}\right)d\omega_{1}\;=-\frac{1}{Z}\sum_{M\mu }\sum_{N\nu }\langle M\mu \left|\frac{\partial H}{\partial \lambda^{l}}\right|N\nu \rangle \langle N\nu \left|\frac{\partial H}{\partial \lambda^{k}}\right|M\mu \rangle \int_{0}^{\beta }e^{-\left(\beta -\omega_{1}\right)E_{N}}e^{-\omega_{1}E_{M}}d\omega_{1}$$

The remaining integral can be solved by recognizing that

87
$$e^{-\beta E_{I}}\int_{0}^{\beta }e^{\omega \left(E_{I}-E_{J}\right)}d\omega =\{\begin{array}{ll} \frac{e^{-\beta E_{J}}}{E_{\mathrm{IJ}}}-\frac{e^{-\beta E_{I}}}{E_{\mathrm{IJ}}}, & E_{I}\neq E_{J}\\ \beta e^{-\beta E_{J}}, & E_{I}=E_{J} \end{array}$$

Here, we introduced the notation *E*_*IJ*_ = *E*_*I*_ – *E*_*J*_ for energy differences. Swapping state labels such that always label *M* occurs in Boltzmann factors (i.e., all Boltzmann factors are equal to *e* ^–β*E*_*M*_^), this leads to the final result

$$\frac{\partial^{2}F}{\partial \lambda^{k}\partial \lambda^{l}}=S^{\left(\mathrm{lk}\right)}\left\{\frac{1}{2}\beta \frac{\partial F}{\partial \lambda^{k}}\frac{\partial F}{\partial \lambda^{l}}-\frac{1}{Z}\underset{M}{\sum }e^{-\beta E_{M}}\left[\underset{\mu \mu^{\prime }}{\sum }\frac{1}{2}\beta \langle M\mu |\frac{\partial H}{\partial \lambda^{k}}|M\mu^{\prime }\rangle \langle M\mu^{\prime }\left|\frac{\partial H}{\partial \lambda^{l}}\right|M\mu \rangle +\underset{\mu }{\sum }\underset{N\neq M,\nu }{\sum }\frac{\langle M\mu \left|\frac{\partial H}{\partial \lambda^{k}}\right|N\nu \rangle \left\langle N\nu \left|\frac{\partial H}{\partial \lambda^{l}}\right|M\mu \right\rangle }{E_{N}-E_{M}}\right]\right\}$$
88

Here, *S* ^(*lk*)^ = 1 + (*lk*) is the symmetrization operator for two parameters. The transposition (*lk*) takes the term to its right and swaps the two parameters *λ* ^*l*^ and *λ* ^*k*^. This equation is (apart from a prefactor) equal to Van Vleck’s equation for the susceptibility tensor and Van den Heuvel and Soncini’s equation for the shielding tensor.^21^

### A.3 Third Derivative of the Free Energy

Starting from eq 85, the third derivative of the free energy is given by (again using Wilcox’s equation)

89
$$\frac{\partial^{3}F}{\partial \lambda^{j}\partial \lambda^{k}\partial \lambda^{l}}=-\beta^{2}\frac{\partial F}{\partial \lambda^{j}}\frac{\partial F}{\partial \lambda^{k}}\frac{\partial F}{\partial \lambda^{l}}+\beta \left(\frac{\partial^{2}F}{\partial \lambda^{j}\partial \lambda^{k}}\frac{\partial F}{\partial \lambda^{l}}+\frac{\partial^{2}F}{\partial \lambda^{j}\partial \lambda^{l}}\frac{\partial F}{\partial \lambda^{k}}+\frac{\partial^{2}F}{\partial \lambda^{k}\partial \lambda^{l}}\frac{\partial F}{\partial \lambda^{j}}\right)+\frac{1}{Z}\left(T_{1}^{\left(3\right)}+T_{2}^{\left(3\right)}\right)$$

where

90
$$T_{1}^{\left(3\right)}=\int_{0}^{\beta }\int_{0}^{\beta -\omega_{1}}\mathrm{tr}\left(\frac{\partial H}{\partial \lambda^{l}}e^{-\left(\beta -\omega_{1}-\omega_{2}\right)H}\frac{\partial H}{\partial \lambda^{j}}e^{-\omega_{2}H}\frac{\partial H}{\partial \lambda^{k}}e^{-\omega_{1}H}\right)d\omega_{2}d\omega_{1}$$

91
$$T_{2}^{\left(3\right)}=\int_{0}^{\beta }\int_{0}^{\omega_{1}}\mathrm{tr}\left(\frac{\partial H}{\partial \lambda^{l}}e^{-\left(\beta -\omega_{1}\right)H}\frac{\partial H}{\partial \lambda^{k}}e^{-\left(\omega_{1}-\omega_{2}\right)H}\frac{\partial H}{\partial \lambda^{j}}e^{-\omega_{2}H}\right)d\omega_{2}d\omega_{1}$$

Under the cyclic permutation (*lkj*) (using cycle notation), the *T*_2_^(3)^ term transforms as

92
$$\left(\mathrm{lkj}\right)T_{2}^{\left(3\right)}=\int_{0}^{\beta }\int_{0}^{\omega_{1}}\mathrm{tr}\left(\frac{\partial H}{\partial \lambda^{k}}e^{-\left(\beta -\omega_{1}\right)H}\frac{\partial H}{\partial \lambda^{j}}e^{-\left(\omega_{1}-\omega_{2}\right)H}\frac{\partial H}{\partial \lambda^{l}}e^{-\omega_{2}H}\right)d\omega_{2}d\omega_{1}=\int_{0}^{\beta }\int_{0}^{\omega_{1}}\mathrm{tr}\left(\frac{\partial H}{\partial \lambda^{l}}e^{-\omega_{2}H}\frac{\partial H}{\partial \lambda^{k}}e^{-\left(\beta -\omega_{1}\right)H}\frac{\partial H}{\partial \lambda^{j}}e^{-\left(\omega_{1}-\omega_{2}\right)H}\right)d\omega_{2}d\omega_{1}=\int_{0}^{\beta }\int_{\beta -\omega_{1}}^{\beta }\mathrm{tr}\left(\frac{\partial H}{\partial \lambda^{l}}e^{-\left(\beta -\omega_{2}^{\prime }\right)H}\frac{\partial H}{\partial \lambda^{k}}e^{-\left(\beta -\omega_{1}\right)H}\frac{\partial H}{\partial \lambda^{j}}e^{-\left(\omega_{1}+\omega_{2}^{\prime }-\beta \right)H}\right)d\omega_{2}^{\prime }d\omega_{1}=\int_{0}^{\beta }\int_{\beta -\omega_{2}^{\prime }}^{\beta }\mathrm{tr}\left(\frac{\partial H}{\partial \lambda^{l}}e^{-\left(\beta -\omega_{2}^{\prime }\right)H}\frac{\partial H}{\partial \lambda^{k}}e^{-\left(\beta -\omega_{1}\right)H}\frac{\partial H}{\partial \lambda^{j}}e^{-\left(\omega_{1}+\omega_{2}^{\prime }-\beta \right)H}\right)d\omega_{1}d\omega_{2}^{\prime }=\int_{0}^{\beta }\int_{0}^{\omega_{2}^{\prime }}\mathrm{tr}\left(\frac{\partial H}{\partial \lambda^{l}}e^{-\left(\beta -\omega_{2}^{\prime }\right)H}\frac{\partial H}{\partial \lambda^{k}}e^{-\left(\omega_{2}^{\prime }-\omega_{1}^{\prime }\right)H}\frac{\partial H}{\partial \lambda^{j}}e^{-\omega_{1}^{\prime }H}\right)d\omega_{1}^{\prime }d\omega_{2}^{\prime }=T_{2}^{\left(3\right)}$$

In the first step, we used the invariance of the expression under cyclic permutations of the operators within the trace. Then, we performed the substitution ω_2_^′^ = β – ω_2_. In the next step, we swapped the order of integration variables by using

93
$$\int_{0}^{\beta }\int_{\beta -\omega_{1}}^{\beta }...d\omega_{2}d\omega_{1}=\int_{0}^{\beta }\int_{\beta -\omega_{2}}^{\beta }...d\omega_{1}d\omega_{2}$$

This identity can be easily understood by visualizing the integration domain as an area in the plane. Finally, we performed the substitution *ω*_1_^′^ = *ω*_2_^′^ – β + *ω*_1_. Since the second cyclic permutation is simply the square of the first, (*ljk*) = (*lkj*)^2^, we obtain that the *T*_2_^(3)^ term is invariant under any cyclic permutation of the three indices *l*, *k*, *j*.

Under the transposition (*jk*), *T*_2_^(3)^ turns into *T*_1_^(3)^:

94
$$\left(\mathrm{kj}\right)T_{2}^{\left(3\right)}=\int_{0}^{\beta }\int_{0}^{\omega_{1}}\mathrm{tr}\left(\frac{\partial H}{\partial \lambda^{l}}e^{-\left(\beta -\omega_{1}\right)H}\frac{\partial H}{\partial \lambda^{j}}e^{-\left(\omega_{1}-\omega_{2}\right)H}\frac{\partial H}{\partial \lambda^{k}}e^{-\omega_{2}H}\right)d\omega_{2}d\omega_{1}=\int_{0}^{\beta }\int_{\omega_{2}}^{\beta }\mathrm{tr}\left(\frac{\partial H}{\partial \lambda^{l}}e^{-\left(\beta -\omega_{1}\right)H}\frac{\partial H}{\partial \lambda^{j}}e^{-\left(\omega_{1}-\omega_{2}\right)H}\frac{\partial H}{\partial \lambda^{k}}e^{-\omega_{2}H}\right)d\omega_{1}d\omega_{2}=\int_{0}^{\beta }\int_{0}^{\beta -\omega_{2}}\mathrm{tr}\left(\frac{\partial H}{\partial \lambda^{l}}e^{-\left(\beta -\omega_{2}-\omega_{1}^{\prime }\right)H}\frac{\partial H}{\partial \lambda^{j}}e^{-\omega_{1}^{\prime }H}\frac{\partial H}{\partial \lambda^{k}}e^{-\omega_{2}H}\right)d\omega_{1}^{\prime }d\omega_{2}=T_{1}^{\left(3\right)}$$

Here, we first swapped the order of integration variables by using

95
$$\int_{0}^{\beta }\int_{0}^{\omega_{1}}...d\omega_{2}d\omega_{1}=\int_{0}^{\beta }\int_{\omega_{2}}^{\beta }...d\omega_{1}d\omega_{2}$$

This identity can again be understood by visualizing the integration domain as an area in the plane. In the next step, we performed the substitution *ω*_1_^′^ = *ω*_1_ – *ω*_2_ to obtain the result. Using eq 94, one can write

96
$$T_{1}^{\left(3\right)}+T_{2}^{\left(3\right)}=\left[1+\left(\mathrm{kj}\right)\right]T_{2}^{\left(3\right)}$$

which means that we only need to explicitly derive an equation for the *T*_2_^(3)^ term. When inserting an eigenbasis of the Hamiltonian in the expression for the *T*_2_^(3)^ term, one obtains

97
$$T_{2}^{\left(3\right)}=\sum_{\mathrm{MNO}}\sum_{\mu \nu o}\left\langle M\mu |\frac{\partial H}{\partial \lambda^{l}}|\mathrm{Oo}\right\rangle \left\langle \mathrm{Oo}|\frac{\partial H}{\partial \lambda^{k}}|N\nu \right\rangle \left\langle N\nu |\frac{\partial H}{\partial \lambda^{j}}|M\mu \right\rangle \times \int_{0}^{\beta }\int_{0}^{\omega_{1}}e^{-\left(\beta -\omega_{1}\right)E_{O}}e^{-\left(\omega_{1}-\omega_{2}\right)E_{N}}e^{-\omega_{2}E_{M}}d\omega_{2}d\omega_{1}=\underset{\mathrm{MNO}}{\sum^{\prime }}\left(\mathrm{ONM}\right)e^{-\beta E_{O}}\int_{0}^{\beta }e^{\omega_{1}E_{O}}e^{-\omega_{1}E_{N}}\int_{0}^{\omega_{1}}e^{\omega_{2}\left(E_{N}-E_{M}\right)}d\omega_{2}d\omega_{1}$$

Here, we introduced the simplified notation

$$\left(\mathrm{ONM}\right)=\langle M\mu |\frac{\partial H}{\partial \lambda^{l}}|\mathrm{Oo}\rangle \langle \mathrm{Oo}|\frac{\partial H}{\partial \lambda^{k}}|N\nu \rangle \langle N\nu \left|\frac{\partial H}{\partial \lambda^{j}}|M\mu \right\rangle$$
98

i.e., the state labels *O*, *N*, *M* indicate which states are in the ket of the three matrix elements (keeping the order of the Hamiltonian derivatives, first *l*, then *k*, then *j*, fixed). Furthermore, the prime on the sum in eq 97 indicates that there is also a summation over degenerate states belonging to each energy level. Now, one can first solve the integration over *ω*_2_ and then the remaining integration over *ω*_1_ using eq 87 and a new type of integral that is given by

99
$$e^{-\beta E_{I}}\int_{0}^{\beta }\omega e^{\omega \left(E_{I}-E_{J}\right)}d\omega =\{\begin{array}{ll} \frac{\beta e^{-\beta E_{J}}}{E_{\mathrm{IJ}}}-\frac{e^{-\beta E_{J}}}{E_{\mathrm{IJ}}^{2}}+\frac{e^{-\beta E_{I}}}{E_{\mathrm{IJ}}^{2}}, & E_{I}\neq E_{J}\\ \frac{1}{2}\beta^{2}e^{-\beta E_{J}}, & E_{I}=E_{J} \end{array}$$

The result is a sum over ten terms. In the next step, state labels are again swapped (like in the case of the 2nd derivative) such that only the label *M* occurs in the Boltzmann factors. In some of the resulting terms, state labels other than *M* may be equal or unequal to *M*. If this is the case, we split those terms further to distinguish the two cases. To give a concrete example, one of the terms where this is the case can be split according to

100
$$-\underset{\begin{array}{c} MNO\\ O\neq M\\ N\neq O \end{array}}{\sum^{\prime }}\frac{\left(\mathrm{MNO}\right)}{E_{\mathrm{MO}}E_{\mathrm{NO}}}e^{-\beta E_{M}}=-\underset{\begin{array}{c} MO\\ O\neq M \end{array}}{\sum^{\prime }}\frac{\left(\mathrm{MMO}\right)}{E_{\mathrm{OM}}^{2}}e^{-\beta E_{M}}-\underset{\begin{array}{c} MNO\\ O\neq M\\ N\neq M\\ N\neq O \end{array}}{\sum^{\prime }}\frac{\left(\mathrm{MNO}\right)}{E_{\mathrm{MO}}E_{\mathrm{NO}}}e^{-\beta E_{M}}$$

On the right-hand side, *N* = *M* in the first term and *N* ≠ *M* in the second term. We know that *T*_2_^(3)^ is overall invariant under cyclic permutations of the three indices *l*, *k*, *j* labeling the derivatives of the Hamiltonian. Before we continue, it is important to comment on how these permutations can be handled in our simplified notation for the numerators. This is probably best illustrated with an example. Acting with the cyclic permutation (*lkj*) on a product of matrix elements in simplified notation, one obtains

101
$$\left(\mathrm{lkj}\right)\left(\mathrm{ONM}\right)=\left(\mathrm{lkj}\right)\left\langle M\mu |\frac{\partial H}{\partial \lambda^{l}}|\mathrm{Oo}\right\rangle \left\langle \mathrm{Oo}|\frac{\partial H}{\partial \lambda^{k}}|N\nu \right\rangle \left\langle N\nu |\frac{\partial H}{\partial \lambda^{j}}|M\mu \right\rangle =\left\langle M\mu |\frac{\partial H}{\partial \lambda^{k}}|\mathrm{Oo}\right\rangle \left\langle \mathrm{Oo}|\frac{\partial H}{\partial \lambda^{j}}|N\nu \right\rangle \left\langle N\nu |\frac{\partial H}{\partial \lambda^{l}}|M\mu \right\rangle =\left\langle N\nu |\frac{\partial H}{\partial \lambda^{l}}|M\mu \right\rangle \left\langle M\mu |\frac{\partial H}{\partial \lambda^{k}}|\mathrm{Oo}\right\rangle \left\langle \mathrm{Oo}|\frac{\partial H}{\partial \lambda^{j}}|N\nu \right\rangle =\left(\mathrm{MON}\right)$$

One can see that in our simplified notation, a cyclic permutation of the indices *l*, *k*, *j* corresponds to an opposite cyclic permutation of the state labels *O*, *N*, *M*. We can now group different terms together whose sum must also individually be invariant under cyclic permutations, e.g., the power of β in a term remains the same after applying a permutation.

There is only one term with a β^2^ prefactor:

102
$$\underset{M}{\sum^{\prime }}\left(\mathrm{MMM}\right)\frac{1}{2}\beta^{2}e^{-\beta E_{M}}$$

It is obvious that this term is invariant under cyclic permutations.

The sum of terms involving a β prefactor is given by

103
$$\underset{\begin{array}{c} MO\\ O\neq M \end{array}}{\sum^{\prime }}\frac{\left(\mathrm{OMM}\right)}{E_{\mathrm{OM}}}\beta e^{-\beta E_{M}}+\underset{\begin{array}{c} MO\\ O\neq M \end{array}}{\sum^{\prime }}\frac{\left(\mathrm{MOM}\right)}{E_{\mathrm{OM}}}\beta e^{-\beta E_{M}}+\underset{\begin{array}{c} MO\\ O\neq M \end{array}}{\sum^{\prime }}\frac{\left(\mathrm{MMO}\right)}{E_{\mathrm{OM}}}\beta e^{-\beta E_{M}}$$

which is also clearly invariant under cyclic permutations.

The sum of terms without β prefactor and including the state label *M* twice is given by

104
$$-\underset{\begin{array}{c} MO\\ O\neq M \end{array}}{\sum^{\prime }}\frac{\left(\mathrm{OMM}\right)}{E_{\mathrm{OM}}^{2}}e^{-\beta E_{M}}-\underset{\begin{array}{c} MO\\ O\neq M \end{array}}{\sum^{\prime }}\frac{\left(\mathrm{MMO}\right)}{E_{\mathrm{OM}}^{2}}e^{-\beta E_{M}}-\underset{\begin{array}{c} MO\\ O\neq M \end{array}}{\sum^{\prime }}\frac{\left(\mathrm{MOM}\right)}{E_{\mathrm{OM}}^{2}}e^{-\beta E_{M}}$$

which is also clearly invariant under cyclic permutations.

All remaining terms are without a β prefactor and include the state label *M* only once. In these terms, *M* can occur in either the first, second, or third position in our simplified notation for the numerators. There is only one term each for *M* in the second and third positions, respectively. Knowing that the *T*_2_^(3)^ term must be overall invariant under cyclic permutations, we *know* that the sum of terms with *M* in the first position must be equal to a cyclic permutation acting on one of the terms with *M* in another position. To make this argument more convincing, we will show this explicitly by combining the three terms with *M* in the first position into a single term:

105
$$-\underset{\begin{array}{c} MNO\\ O\neq M\\ N\neq M\\ N\neq O \end{array}}{\sum^{\prime }}\frac{\left(\mathrm{MNO}\right)}{E_{\mathrm{MO}}E_{\mathrm{NO}}}e^{-\beta E_{M}}+\underset{\begin{array}{c} MNO\\ O\neq M\\ N\neq M\\ N\neq O \end{array}}{\sum^{\prime }}\frac{\left(\mathrm{MNO}\right)}{E_{\mathrm{NO}}E_{\mathrm{MN}}}e^{-\beta E_{M}}+\underset{\begin{array}{c} MO\\ O\neq M \end{array}}{\sum^{\prime }}\frac{\left(\mathrm{MOO}\right)}{E_{\mathrm{OM}}^{2}}e^{-\beta E_{M}}=-\underset{\begin{array}{c} MNO\\ O\neq M\\ N\neq M\\ N\neq O \end{array}}{\sum^{\prime }}\frac{\left(\mathrm{MNO}\right)}{E_{\mathrm{MO}}E_{\mathrm{NO}}E_{\mathrm{MN}}}\times \left(E_{\mathrm{MN}}-E_{\mathrm{MO}}\right)e^{-\beta E_{M}}+\underset{\begin{array}{c} MO\\ O\neq M \end{array}}{\sum^{\prime }}\frac{\left(\mathrm{MOO}\right)}{E_{\mathrm{OM}}^{2}}e^{-\beta E_{M}}=\underset{\begin{array}{c} MNO\\ O\neq M\\ N\neq M\\ N\neq O \end{array}}{\sum^{\prime }}\frac{\left(\mathrm{MNO}\right)}{E_{\mathrm{NM}}E_{\mathrm{OM}}}e^{-\beta E_{M}}+\underset{\begin{array}{c} MO\\ O\neq M \end{array}}{\sum^{\prime }}\frac{\left(\mathrm{MOO}\right)}{E_{\mathrm{OM}}^{2}}e^{-\beta E_{M}}=\underset{\begin{array}{c} MNO\\ O\neq M\\ N\neq M \end{array}}{\sum^{\prime }}\frac{\left(\mathrm{MNO}\right)}{E_{\mathrm{NM}}E_{\mathrm{OM}}}e^{-\beta E_{M}}$$

In the first step, we combined the first two terms by choosing a common denominator. Afterwards, we cancel the resulting *E*_*MN*_ – *E*_*MO*_ = *E*_*ON*_ with the *E*_*NO*_ in the denominator. Finally, the two cases *N* ≠ *O* and *N* = *O* are combined into a single term. This shows that the sum of all terms without a β prefactor and including the state label *M* only once is given by

106
$$\underset{\begin{array}{c} MNO\\ O\neq M\\ N\neq M \end{array}}{\sum^{\prime }}\frac{\left(\mathrm{MNO}\right)}{E_{\mathrm{NM}}E_{\mathrm{OM}}}e^{-\beta E_{M}}+\underset{\begin{array}{c} MNO\\ O\neq M\\ N\neq M \end{array}}{\sum^{\prime }}\frac{\left(\mathrm{OMN}\right)}{E_{\mathrm{NM}}E_{\mathrm{OM}}}e^{-\beta E_{M}}+\underset{\begin{array}{c} MNO\\ O\neq M\\ N\neq M \end{array}}{\sum^{\prime }}\frac{\left(\mathrm{NOM}\right)}{E_{\mathrm{NM}}E_{\mathrm{OM}}}e^{-\beta E_{M}}$$

which is, as expected, invariant under cyclic permutations.

Combining all these terms leads to

107
$$T_{2}^{\left(3\right)}=S_{\mathrm{circ}}^{\left(\mathrm{lkj}\right)}\left[\underset{M}{\sum^{\prime }}\left(\mathrm{MMM}\right)\frac{1}{6}\beta^{2}e^{-\beta E_{M}}+\underset{\begin{array}{c} MO\\ O\neq M \end{array}}{\sum^{\prime }}\frac{\left(\mathrm{OMM}\right)}{E_{\mathrm{OM}}}\beta e^{-\beta E_{M}}-\underset{\begin{array}{c} MO\\ O\neq M \end{array}}{\sum^{\prime }}\frac{\left(\mathrm{OMM}\right)}{E_{\mathrm{OM}}^{2}}e^{-\beta E_{M}}+\underset{\begin{array}{c} MNO\\ O\neq M\\ N\neq M \end{array}}{\sum^{\prime }}\frac{\left(\mathrm{ONM}\right)}{E_{\mathrm{NM}}E_{\mathrm{OM}}}e^{-\beta E_{M}}\right]$$

where *S*_circ_^(*lkj*)^ = 1 + (*lkj*) + (*ljk*) is the sum of circular shifts, i.e., the symmetrizer with respect to the subgroup of order 3 (of the symmetric group *S*_3_(*lkj*)) consisting of circular shifts. Since each transposition can be written as the product of (*kj*) with a circular shift, it follows that

108
$$\left[1+\left(\mathrm{kj}\right)\right]S_{\mathrm{circ}}^{\left(\mathrm{lkj}\right)}=S^{\left(\mathrm{lkj}\right)}=\sum_{\pi \in S_{3}\left(\mathrm{lkj}\right)}\pi$$

yields the full symmetrizer *S* ^(*lkj*)^. We can now write down the final equation for the third derivative of the free energy, where we went back from the simplified notation to full notation,

$$\frac{\partial^{3}F}{\partial \lambda^{j}\partial \lambda^{k}\partial \lambda^{l}}=S^{\left(\mathrm{lkj}\right)}\left\{-\frac{1}{6}\beta^{2}\frac{\partial F}{\partial \lambda^{l}}\frac{\partial F}{\partial \lambda^{k}}\frac{\partial F}{\partial \lambda^{j}}+\frac{1}{2}\beta \frac{\partial F}{\partial \lambda^{l}}\frac{\partial^{2}F}{\partial \lambda^{k}\partial \lambda^{j}}+\frac{1}{Z}\underset{M}{\sum }e^{-\beta E_{M}}\left[\underset{\mu \mu^{\prime }\mu^{\prime \prime }}{\sum }\frac{1}{6}\beta^{2}\left\langle M\mu \left|\frac{\partial H}{\partial \lambda^{l}}\right|M\mu^{\prime \prime }\right\rangle \left\langle M\mu^{\prime \prime }\left|\frac{\partial H}{\partial \lambda^{k}}\right|M\mu^{\prime }\right\rangle \left\langle M\mu^{\prime }\left|\frac{\partial H}{\partial \lambda^{j}}\right|M\mu \right\rangle +\underset{\mu \mu^{\prime }}{\sum }\underset{O\neq M,o}{\sum }\beta \frac{\langle M\mu \left|\frac{\partial H}{\partial \lambda^{l}}\right|\mathrm{Oo}\rangle \left\langle \mathrm{Oo}\left|\frac{\partial H}{\partial \lambda^{k}}\right|M\mu^{\prime }\right\rangle \left\langle M\mu^{\prime }\left|\frac{\partial H}{\partial \lambda^{j}}\right|M\mu \right\rangle }{E_{O}-E_{M}}-\underset{\mu \mu^{\prime }}{\sum }\underset{O\neq M,o}{\sum }\frac{\langle M\mu \left|\frac{\partial H}{\partial \lambda^{l}}\right|\mathrm{Oo}\rangle \left\langle \mathrm{Oo}\left|\frac{\partial H}{\partial \lambda^{k}}\right|M\mu^{\prime }\right\rangle \left\langle M\mu^{\prime }\left|\frac{\partial H}{\partial \lambda^{j}}\right|M\mu \right\rangle }{{\left(E_{O}-E_{M}\right)}^{2}}+\underset{\mu }{\sum }\underset{O\neq M,o}{\sum }\underset{N\neq M,\nu }{\sum }\frac{\langle M\mu \left|\frac{\partial H}{\partial \lambda^{l}}\right|\mathrm{Oo}\rangle \left\langle \mathrm{Oo}\left|\frac{\partial H}{\partial \lambda^{k}}\right|N\nu \right\rangle \left\langle N\nu \left|\frac{\partial H}{\partial \lambda^{j}}M\mu \right|\right\rangle }{\left(E_{N}-E_{M}\right)\left(E_{O}-E_{M}\right)}\right]\right\}$$
109

### A.4 Fourth Derivative of the Free Energy

By taking the derivative of eq 89 (with *T*_1_^(3)^ + *T*_2_^(3)^ substituted according to eq 96) with respect to *λ* ^*i*^, one arrives at

110
$$\frac{\partial^{4}F}{\partial \lambda^{i}\partial \lambda^{j}\partial \lambda^{k}\partial \lambda^{l}}=\beta^{3}\frac{\partial F}{\partial \lambda^{i}}\frac{\partial F}{\partial \lambda^{j}}\frac{\partial F}{\partial \lambda^{k}}\frac{\partial F}{\partial \lambda^{l}}-\beta^{2}\left[\frac{\partial^{2}F}{\partial \lambda^{i}\partial \lambda^{j}}\frac{\partial F}{\partial \lambda^{k}}\frac{\partial F}{\partial \lambda^{l}}+\frac{\partial^{2}F}{\partial \lambda^{i}\partial \lambda^{k}}\frac{\partial F}{\partial \lambda^{j}}\frac{\partial F}{\partial \lambda^{l}}+\frac{\partial^{2}F}{\partial \lambda^{i}\partial \lambda^{l}}\frac{\partial F}{\partial \lambda^{j}}\frac{\partial F}{\partial \lambda^{k}}+\frac{\partial^{2}F}{\partial \lambda^{j}\partial \lambda^{k}}\frac{\partial F}{\partial \lambda^{i}}\frac{\partial F}{\partial \lambda^{l}}+\frac{\partial^{2}F}{\partial \lambda^{j}\partial \lambda^{l}}\frac{\partial F}{\partial \lambda^{i}}\frac{\partial F}{\partial \lambda^{k}}+\frac{\partial^{2}F}{\partial \lambda^{k}\partial \lambda^{l}}\frac{\partial F}{\partial \lambda^{i}}\frac{\partial F}{\partial \lambda^{j}}\right]+\beta \left[\frac{\partial^{3}F}{\partial \lambda^{i}\partial \lambda^{j}\partial \lambda^{k}}\frac{\partial F}{\partial \lambda^{l}}+\frac{\partial^{3}F}{\partial \lambda^{i}\partial \lambda^{j}\partial \lambda^{l}}\frac{\partial F}{\partial \lambda^{k}}+\frac{\partial^{3}F}{\partial \lambda^{i}\partial \lambda^{k}\partial \lambda^{l}}\frac{\partial F}{\partial \lambda^{j}}+\frac{\partial^{3}F}{\partial \lambda^{j}\partial \lambda^{k}\partial \lambda^{l}}\frac{\partial F}{\partial \lambda^{i}}\right]+\beta [\frac{\partial^{2}F}{\partial \lambda^{j}\partial \lambda^{k}}\frac{\partial^{2}F}{\partial \lambda^{i}\partial \lambda^{l}}+\frac{\partial^{2}F}{\partial \lambda^{j}\partial \lambda^{l}}\frac{\partial^{2}F}{\partial \lambda^{i}\partial \lambda^{k}}+\frac{\partial^{2}F}{\partial \lambda^{k}\partial \lambda^{l}}\frac{\partial^{2}F}{\partial \lambda^{i}\partial \lambda^{j}}]+\left[1+\left(\mathrm{kj}\right)\right]\frac{1}{Z}\frac{\partial T_{2}^{\left(3\right)}}{\partial \lambda^{i}}$$

Using Wilcox’s equation [eq 75], the derivative of the *T*_2_^(3)^ term can be written as

111
$$\frac{\partial T_{2}^{\left(3\right)}}{\partial \lambda^{i}}=-\left(T_{1}^{\left(4\right)}+T_{2}^{\left(4\right)}+T_{3}^{\left(4\right)}\right)$$

where the individual terms are given by

112
$$T_{1}^{\left(4\right)}=\int_{0}^{\beta }\int_{0}^{\omega_{1}}\int_{0}^{\beta -\omega_{1}}\mathrm{tr}\left(\frac{\partial H}{\partial \lambda^{l}}e^{-\left(\beta -\omega_{1}-\omega_{3}\right)H}\frac{\partial H}{\partial \lambda^{i}}e^{-\omega_{3}H}\frac{\partial H}{\partial \lambda^{k}}e^{-\left(\omega_{1}-\omega_{2}\right)H}\frac{\partial H}{\partial \lambda^{j}}e^{-\omega_{2}H}\right)d\omega_{3}d\omega_{2}d\omega_{1}$$

113
$$T_{2}^{\left(4\right)}=\int_{0}^{\beta }\int_{0}^{\omega_{1}}\int_{0}^{\omega_{1}-\omega_{2}}\mathrm{tr}\left(\frac{\partial H}{\partial \lambda^{l}}e^{-\left(\beta -\omega_{1}\right)H}\frac{\partial H}{\partial \lambda^{k}}e^{-\left(\omega_{1}-\omega_{2}-\omega_{3}\right)H}\frac{\partial H}{\partial \lambda^{i}}e^{-\omega_{3}H}\frac{\partial H}{\partial \lambda^{j}}e^{-\omega_{2}H}\right)d\omega_{3}d\omega_{2}d\omega_{1}$$

114
$$T_{3}^{\left(4\right)}=\int_{0}^{\beta }\int_{0}^{\omega_{1}}\int_{0}^{\omega_{2}}\mathrm{tr}\left(\frac{\partial H}{\partial \lambda^{l}}e^{-\left(\beta -\omega_{1}\right)H}\frac{\partial H}{\partial \lambda^{k}}e^{-\left(\omega_{1}-\omega_{2}\right)H}\frac{\partial H}{\partial \lambda^{j}}e^{-\left(\omega_{2}-\omega_{3}\right)H}\frac{\partial H}{\partial \lambda^{i}}e^{-\omega_{3}H}\right)d\omega_{3}d\omega_{2}d\omega_{1}$$

We anticipate that, in analogy to our derivation of the third derivative, the *T*_3_^(4)^ term is invariant under the subgroup of order 4 generated by the cyclic permutation (*lkji*) (the group of circular shifts), and that the *T*_1_^(4)^ and *T*_2_^(4)^ terms can be generated from *T*_3_^(4)^ by applying certain permutations of the indices *l*, *k*, *j*, *i*. Since the trace is invariant under circular shifts of a product of operators, one gets

115
$$\left(\mathrm{lkji}\right)T_{3}^{\left(4\right)}=\int_{0}^{\beta }\int_{0}^{\omega_{1}}\int_{0}^{\omega_{2}}\mathrm{tr}\left(\frac{\partial H}{\partial \lambda^{l}}e^{-\omega_{3}H}\frac{\partial H}{\partial \lambda^{k}}e^{-\left(\beta -\omega_{1}\right)H}\frac{\partial H}{\partial \lambda^{j}}e^{-\left(\omega_{1}-\omega_{2}\right)H}\frac{\partial H}{\partial \lambda^{i}}e^{-\left(\omega_{2}-\omega_{3}\right)H}\right)d\omega_{3}d\omega_{2}d\omega_{1}$$

Comparing coefficients in the exponentials, one sees that we must make substitutions such that

116
$${\mathrm{\omega ̃}}_{1}=\beta -\omega_{3}$$

117
$${\mathrm{\omega ̃}}_{2}=\omega_{1}-\omega_{3}$$

118
$${\mathrm{\omega ̃}}_{3}=\omega_{2}-\omega_{3}$$

We can achieve this by the following sequence of substitutions outlined in Table 3: First, we swap labels 2 and 3 and then we swap the (new) labels 1 and 2. Associated with each swap comes also a change in the order of integration, with the integration over index 3 always being innermost and the integration over index 1 always being outermost. Finally, we can perform the three substitutions in the last column of Table 3. Executing these steps, from eq 115 one obtains

119
$$\left(\mathrm{lkji}\right)T_{3}^{\left(4\right)}=\int_{0}^{\beta }\int_{0}^{\omega_{1}^{\prime }}\int_{\omega_{2}^{\prime }}^{\omega_{1}^{\prime }}\mathrm{tr}\left(\frac{\partial H}{\partial \lambda^{l}}e^{-\omega_{2}^{\prime }H}\frac{\partial H}{\partial \lambda^{k}}e^{-\left(\beta -\omega_{1}^{\prime }\right)H}\frac{\partial H}{\partial \lambda^{j}}e^{-\left(\omega_{1}^{\prime }-\omega_{3}^{\prime }\right)H}\frac{\partial H}{\partial \lambda^{i}}e^{-\left(\omega_{3}^{\prime }-\omega_{2}^{\prime }\right)H}\right)d\omega_{3}^{\prime }d\omega_{2}^{\prime }d\omega_{1}^{\prime }=\int_{0}^{\beta }\int_{\omega_{1}^{\prime \prime }}^{\beta }\int_{\omega_{1}^{\prime \prime }}^{\omega_{2}^{\prime \prime }}\mathrm{tr}\left(\frac{\partial H}{\partial \lambda^{l}}e^{-\omega_{1}^{\prime \prime }H}\frac{\partial H}{\partial \lambda^{k}}e^{-\left(\beta -\omega_{2}^{\prime \prime }\right)H}\frac{\partial H}{\partial \lambda^{j}}e^{-\left(\omega_{2}^{\prime \prime }-\omega_{3}^{\prime \prime }\right)H}\frac{\partial H}{\partial \lambda^{i}}e^{-\left(\omega_{3}^{\prime \prime }-\omega_{1}^{\prime \prime }\right)H}\right)d\omega_{3}^{\prime \prime }d\omega_{2}^{\prime \prime }d\omega_{1}^{\prime \prime }=\int_{0}^{\beta }\int_{\omega_{1}^{\prime \prime }}^{\beta }\int_{0}^{\omega_{2}^{\prime \prime }-\omega_{1}^{\prime \prime }}\mathrm{tr}\left(\frac{\partial H}{\partial \lambda^{l}}e^{-\omega_{1}^{\prime \prime }H}\frac{\partial H}{\partial \lambda^{k}}e^{-\left(\beta -\omega_{2}^{\prime \prime }\right)H}\frac{\partial H}{\partial \lambda^{j}}e^{-\left(\omega_{2}^{\prime \prime }-{\mathrm{\omega ̃}}_{3}-\omega_{1}^{\prime \prime }\right)H}\frac{\partial H}{\partial \lambda^{i}}e^{-{\mathrm{\omega ̃}}_{3}H}\right)d{\mathrm{\omega ̃}}_{3}d\omega_{2}^{\prime \prime }d\omega_{1}^{\prime \prime }=\int_{0}^{\beta }\int_{0}^{\beta -\omega_{1}^{\prime \prime }}\int_{0}^{{\mathrm{\omega ̃}}_{2}}\mathrm{tr}\left(\frac{\partial H}{\partial \lambda^{l}}e^{-\omega_{1}^{\prime \prime }H}\frac{\partial H}{\partial \lambda^{k}}e^{-\left(\beta -{\mathrm{\omega ̃}}_{2}-\omega_{1}^{\prime \prime }\right)H}\frac{\partial H}{\partial \lambda^{j}}e^{-\left({\mathrm{\omega ̃}}_{2}-{\mathrm{\omega ̃}}_{3}\right)H}\frac{\partial H}{\partial \lambda^{i}}e^{-{\mathrm{\omega ̃}}_{3}H}\right)d{\mathrm{\omega ̃}}_{3}d{\mathrm{\omega ̃}}_{2}d\omega_{1}^{\prime \prime }=\int_{0}^{\beta }\int_{0}^{{\mathrm{\omega ̃}}_{1}}\int_{0}^{{\mathrm{\omega ̃}}_{2}}\mathrm{tr}\left(\frac{\partial H}{\partial \lambda^{l}}e^{-\left(\beta -{\mathrm{\omega ̃}}_{1}\right)H}\frac{\partial H}{\partial \lambda^{k}}e^{-\left({\mathrm{\omega ̃}}_{1}-{\mathrm{\omega ̃}}_{2}\right)H}\frac{\partial H}{\partial \lambda^{j}}e^{-\left({\mathrm{\omega ̃}}_{2}-{\mathrm{\omega ̃}}_{3}\right)H}\frac{\partial H}{\partial \lambda^{i}}e^{-{\mathrm{\omega ̃}}_{3}H}\right)d{\mathrm{\omega ̃}}_{3}d{\mathrm{\omega ̃}}_{2}d{\mathrm{\omega ̃}}_{1}=T_{3}^{\left(4\right)}$$

This shows that *T*_3_^(4)^ is indeed, as anticipated, invariant under the permutation (*lkji*) and therefore also under the whole subgroup of circular shifts generated by it.

**Table 3:** Sequence of Variable Substitutions for Showing That (*lkji*)*T*_3_^(4)^ = *T*_3_^(4)^

first	second	third
ω_1_^′^ = ω_1_	ω_1_^″^ = ω_2_^′^	ω̃_1_ = β – ω_1_^″^
ω_2_^′^ = ω_3_	ω_2_^″^ = ω_1_^′^	ω̃_2_ = ω_2_^″^ – ω_1_^″^
ω_3_^′^ = ω_2_	ω_3_^″^ = ω_3_^′^	ω̃_3_ = ω_3_^″^ – ω_1_^″^

**Table 4:** Sequence of Variable Substitutions for Showing That (*kij*)*T*_3_^(4)^ = *T*_1_^(4)^

first	second	third
ω_1_^′^ = ω_2_	ω_1_^″^ = ω_1_^′^	ω̃_1_ = ω_1_^″^
ω_2_^′^ = ω_1_	ω_2_^″^ = ω_3_^′^	ω̃_2_ = ω_2_^″^
ω_3_^′^ = ω_3_	ω_3_^″^ = ω_2_^′^	ω̃_3_ = ω_3_^″^ – ω_1_^″^

The permutation (*ij*) generates *T*_2_^(4)^,

120
$$\left(\mathrm{ij}\right)T_{3}^{\left(4\right)}=T_{2}^{\left(4\right)}$$

We skip the detailed derivation here since it is basically identical to the derivation of eq 94. Finally, the permutation (*kij*) brings the Hamiltonian derivatives in *T*_3_^(4)^ into the order they occur in *T*_1_^(4)^, so we expect that the result is equal to *T*_1_^(4)^. Comparing coefficients in the exponentials, one sees that we must make substitutions such that

121
$${\mathrm{\omega ̃}}_{1}=\omega_{2}$$

122
$${\mathrm{\omega ̃}}_{2}=\omega_{3}$$

123
$${\mathrm{\omega ̃}}_{3}=\omega_{1}-\omega_{2}$$

To achieve this, we perform the sequence of substitutions summarized in Table 4: we first swap labels 1 and 2 and then we swap the (new) labels 2 and 3. Again, associated with each swap comes also a change in the order of integration. Finally, we perform the single substitution *ω̃*_3_ = *ω*_3_^″^ – *ω*_1_^″^ (and the other two variables stay unchanged). Applying these steps leads to

$$\left(\mathrm{kij}\right)T_{3}^{\left(4\right)}=\int_{0}^{\beta }\int_{0}^{\omega_{1}}\int_{0}^{\omega_{2}}\mathrm{tr}\left(\frac{\partial H}{\partial \lambda^{l}}e^{-\left(\beta -\omega_{1}\right)H}\frac{\partial H}{\partial \lambda^{i}}e^{-\left(\omega_{1}-\omega_{2}\right)H}\frac{\partial H}{\partial \lambda^{k}}e^{-\left(\omega_{2}-\omega_{3}\right)H}\frac{\partial H}{\partial \lambda^{j}}e^{-\omega_{3}H}\right)d\omega_{3}d\omega_{2}d\omega_{1}=\int_{0}^{\beta }\int_{\omega_{1}^{\prime }}^{\beta }\int_{0}^{\omega_{1}^{\prime }}\mathrm{tr}\left(\frac{\partial H}{\partial \lambda^{l}}e^{-\left(\beta -\omega_{2}^{\prime }\right)H}\frac{\partial H}{\partial \lambda^{i}}e^{-\left(\omega_{2}^{\prime }-\omega_{1}^{\prime }\right)H}\frac{\partial H}{\partial \lambda^{k}}e^{-\left(\omega_{1}^{\prime }-\omega_{3}^{\prime }\right)H}\frac{\partial H}{\partial \lambda^{j}}e^{-\omega_{3}^{\prime }H}\right)d\omega_{3}^{\prime }d\omega_{2}^{\prime }d\omega_{1}^{\prime }=\int_{0}^{\beta }\int_{0}^{\omega_{1}^{\prime \prime }}\int_{\omega_{1}^{\prime \prime }}^{\beta }\mathrm{tr}\left(\frac{\partial H}{\partial \lambda^{l}}e^{-\left(\beta -\omega_{3}^{\prime \prime }\right)H}\frac{\partial H}{\partial \lambda^{i}}e^{-\left(\omega_{3}^{\prime \prime }-\omega_{1}^{\prime \prime }\right)H}\frac{\partial H}{\partial \lambda^{k}}e^{-\left(\omega_{1}^{\prime \prime }-\omega_{2}^{\prime \prime }\right)H}\frac{\partial H}{\partial \lambda^{j}}e^{-\omega_{2}^{\prime \prime }H}\right)d\omega_{3}^{\prime \prime }d\omega_{2}^{\prime \prime }d\omega_{1}^{\prime \prime }=\int_{0}^{\beta }\int_{0}^{{\mathrm{\omega ̃}}_{1}}\int_{0}^{\beta -{\mathrm{\omega ̃}}_{1}}\mathrm{tr}\left(\frac{\partial H}{\partial \lambda^{l}}e^{-\left(\beta -{\mathrm{\omega ̃}}_{3}-{\mathrm{\omega ̃}}_{1}\right)H}\frac{\partial H}{\partial \lambda^{i}}e^{-{\mathrm{\omega ̃}}_{3}H}\frac{\partial H}{\partial \lambda^{k}}e^{-\left({\mathrm{\omega ̃}}_{1}-{\mathrm{\omega ̃}}_{2}\right)H}\frac{\partial H}{\partial \lambda^{j}}e^{-{\mathrm{\omega ̃}}_{2}H}\right)d{\mathrm{\omega ̃}}_{3}d{\mathrm{\omega ̃}}_{2}d{\mathrm{\omega ̃}}_{1}=T_{1}^{\left(4\right)}$$
124

It follows that one can write

125
$$\left[1+\left(\mathrm{kj}\right)\right]\frac{1}{Z}\frac{\partial T_{2}^{\left(3\right)}}{\partial \lambda^{i}}=-\frac{1}{Z}\left[1+\left(\mathrm{kj}\right)\right]\left[1+\left(\mathrm{ij}\right)+\left(\mathrm{kij}\right)\right]T_{3}^{\left(4\right)}=-\frac{1}{Z}S^{\left(\mathrm{kji}\right)}T_{3}^{\left(4\right)}$$

where *S* ^(*kji*)^ is the symmetrizer over the three indices *k*, *j*, *i*. The final task is now to find a sum-over-states expression for *T*_3_^(4)^. The derivation is much more complex than for *T*_2_^(3)^, but otherwise proceeds along exactly the same lines. Therefore, we do not go into the same level of detail here. After inserting an eigenbasis of the Hamiltonian, one obtains (again making use of the simplified notation introduced in the previous section)

126
$$T_{3}^{\left(4\right)}=\underset{\mathrm{MPON}}{\sum^{\prime }}\left(\mathrm{PONM}\right)e^{-\beta E_{P}}\int_{0}^{\beta }e^{\omega_{1}E_{P}}e^{-\omega_{1}E_{O}}\int_{0}^{\omega_{1}}e^{\omega_{2}E_{O}}e^{-\omega_{2}E_{N}}\int_{0}^{\omega_{2}}e^{\omega_{3}\left(E_{N}-E_{M}\right)}d\omega_{3}d\omega_{2}d\omega_{1}$$

Using eqs 87 and 99 as well as a new type of integral given by

127
$$e^{-\beta E_{I}}\int_{0}^{\beta }\frac{1}{2}\omega^{2}e^{\omega \left(E_{I}-E_{J}\right)}d\omega =\{\begin{array}{ll} \frac{\frac{1}{2}\beta^{2}e^{-\beta E_{J}}}{E_{\mathrm{IJ}}}-\frac{\beta e^{-\beta E_{J}}}{E_{\mathrm{IJ}}^{2}}+\frac{e^{-\beta E_{J}}}{E_{\mathrm{IJ}}^{3}}-\frac{e^{-\beta E_{I}}}{E_{\mathrm{IJ}}^{3}}, & E_{I}\neq E_{J}\\ \frac{1}{6}\beta^{3}e^{-\beta E_{J}}, & E_{I}=E_{J} \end{array}$$

we can first perform the integration over *ω*_3_, then over *ω*_2_, and finally over *ω*_1_. The result is a sum over 35 different terms. Next, we again swap state labels such that only label *M* occurs in the Boltzmann factors, and finally, we again split terms such that all state labels other than *M* only run over states different than *M*. At this stage, one can combine the terms into groups that must be individually invariant under the circular shift group of four indices. The result is

128
$$T_{3}^{\left(4\right)}=S_{\mathrm{circ}}^{\left(\mathrm{lkji}\right)}\left[\underset{M}{\sum^{\prime }}\left(\mathrm{MMMM}\right)\frac{1}{24}\beta^{3}e^{-\beta E_{M}}+\underset{\begin{array}{c} MP\\ P\neq M \end{array}}{\sum^{\prime }}\frac{\left(\mathrm{PMMM}\right)}{E_{\mathrm{PM}}}\frac{1}{2}\beta^{2}e^{-\beta E_{M}}-\underset{\begin{array}{c} MP\\ P\neq M \end{array}}{\sum^{\prime }}\frac{\left(\mathrm{PMMM}\right)}{E_{\mathrm{PM}}^{2}}\beta e^{-\beta E_{M}}+\frac{1}{2}\underset{\begin{array}{c} MPN\\ N\neq M\\ P\neq M \end{array}}{\sum^{\prime }}\frac{\left(\mathrm{PMNM}\right)}{E_{\mathrm{NM}}E_{\mathrm{PM}}}\beta e^{-\beta E_{M}}+\frac{1}{2}\underset{\begin{array}{c} MPN\\ N\neq M\\ P\neq M \end{array}}{\sum^{\prime }}\frac{\left(\mathrm{PNMM}\right)}{E_{\mathrm{NM}}E_{\mathrm{PM}}}\beta e^{-\beta E_{M}}+\underset{\begin{array}{c} MP\\ P\neq M \end{array}}{\sum^{\prime }}\frac{\left(\mathrm{PMMM}\right)}{E_{\mathrm{PM}}^{3}}e^{-\beta E_{M}}-\underset{\begin{array}{c} MPN\\ N\neq M\\ P\neq M \end{array}}{\sum^{\prime }}\frac{\left(\mathrm{PMNM}\right)}{E_{\mathrm{NM}}E_{\mathrm{PM}}^{2}}e^{-\beta E_{M}}-\underset{\begin{array}{c} MPN\\ N\neq M\\ P\neq M \end{array}}{\sum^{\prime }}\frac{\left(\mathrm{PNMM}\right)}{E_{\mathrm{NM}}E_{\mathrm{PM}}^{2}}e^{-\beta E_{M}}-\underset{\begin{array}{c} MPN\\ N\neq M\\ P\neq M \end{array}}{\sum^{\prime }}\frac{\left(\mathrm{PNMM}\right)}{E_{\mathrm{PM}}E_{\mathrm{NM}}^{2}}e^{-\beta E_{M}}+\underset{\begin{array}{c} MPON\\ N\neq M\\ P\neq M\\ O\neq M \end{array}}{\sum^{\prime }}\frac{\left(\mathrm{NOPM}\right)}{E_{\mathrm{NM}}E_{\mathrm{PM}}E_{\mathrm{OM}}}e^{-\beta E_{M}}\right]$$

Here, *S*_circ_^(*lkji*)^ = 1 + (*lkji*) + (*lkji*)^2^ + (*lkji*)^3^ is the symmetrizer for the circular shift group of four indices. The elements of *S* ^(*kji*)^ generate the six left cosets of this subgroup, such that *S* ^(*kji)*^ *S*_circ_^(*lkji*)^ = *S* ^(*lkji*)^ is the full symmetrizer of the permutation group of four indices. Out of the 10 terms in eq 128, terms 8 and 9 are each other’s complex conjugate, i.e., only one of them needs to be calculated explicitly. The complete fourth derivative of the free energy can be written as (passing back from simplified to full notation)

$$\frac{\partial^{4}F}{\partial \lambda^{i}\partial \lambda^{j}\partial \lambda^{k}\partial \lambda^{l}}=S^{\left(\mathrm{lkji}\right)}\left\{\frac{1}{24}\beta^{3}\frac{\partial F}{\partial \lambda^{i}}\frac{\partial F}{\partial \lambda^{j}}\frac{\partial F}{\partial \lambda^{k}}\frac{\partial F}{\partial \lambda^{l}}-\frac{1}{4}\beta^{2}\frac{\partial F}{\partial \lambda^{i}}\frac{\partial F}{\partial \lambda^{j}}\frac{\partial F}{\partial \lambda^{k}\partial \lambda^{l}}+\frac{1}{6}\beta \frac{\partial F}{\partial \lambda^{i}}\frac{\partial^{3}F}{\partial \lambda^{j}\partial \lambda^{k}\partial \lambda^{l}}+\frac{1}{8}\beta \frac{\partial^{2}F}{\partial \lambda^{i}\partial \lambda^{j}}\frac{\partial^{2}F}{\partial \lambda^{k}\partial \lambda^{l}}-\frac{1}{Z}\underset{M}{\sum }e^{-\beta E_{M}}\left[\underset{\mu \mu^{\prime }\mu^{\prime \prime }\mu^{\prime \prime \prime }}{\sum }\frac{1}{24}\beta^{3}\left\langle M\mu \left|\frac{\partial H}{\partial \lambda^{l}}\right|M\mu^{\prime \prime \prime }\right\rangle \left\langle M\mu^{\prime \prime \prime }\left|\frac{\partial H}{\partial \lambda^{k}}\right|M\mu^{\prime \prime }\right\rangle \left\langle M\mu^{\prime \prime }\left|\frac{\partial H}{\partial \lambda^{j}}\right|M\mu^{\prime }\right\rangle \left\langle M\mu^{\prime }\left|\frac{\partial H}{\partial \lambda^{i}}\right|M\mu \right\rangle +\underset{\mu \mu^{\prime }\mu^{\prime \prime }}{\sum }\underset{P\neq M,\pi }{\sum }\frac{1}{2}\beta^{2}\frac{\langle M\mu \left|\frac{\partial H}{\partial \lambda^{l}}\right|P\pi \rangle \left\langle P\pi \left|\frac{\partial H}{\partial \lambda^{k}}\right|M\mu^{\prime \prime }\right\rangle \left\langle M\mu^{\prime \prime }\left|\frac{\partial H}{\partial \lambda^{j}}\right|M\mu^{\prime }\right\rangle \left\langle M\mu^{\prime }\left|\frac{\partial H}{\partial \lambda^{i}}\right|M\mu \right\rangle }{E_{P}-E_{M}}-\underset{\mu \mu^{\prime }\mu^{\prime \prime }}{\sum }\underset{P\neq M,\pi }{\sum }\beta \frac{\langle M\mu \left|\frac{\partial H}{\partial \lambda^{l}}\right|P\pi \rangle \left\langle P\pi \left|\frac{\partial H}{\partial \lambda^{k}}\right|M\mu^{\prime \prime }\right\rangle \left\langle M\mu^{\prime \prime }\left|\frac{\partial H}{\partial \lambda^{j}}\right|M\mu^{\prime }\right\rangle \left\langle M\mu^{\prime }\left|\frac{\partial H}{\partial \lambda^{i}}\right|M\mu \right\rangle }{{\left(E_{P}-E_{M}\right)}^{2}}+\underset{\mu \mu^{\prime }\mu^{\prime \prime }}{\sum }\underset{P\neq M,\pi }{\sum }\frac{\langle M\mu \left|\frac{\partial H}{\partial \lambda^{l}}\right|P\pi \rangle \left\langle P\pi \left|\frac{\partial H}{\partial \lambda^{k}}\right|M\mu^{\prime \prime }\right\rangle \left\langle M\mu^{\prime \prime }\left|\frac{\partial H}{\partial \lambda^{j}}\right|M\mu^{\prime }\right\rangle \left\langle M\mu^{\prime }\left|\frac{\partial H}{\partial \lambda^{i}}\right|M\mu \right\rangle }{{\left(E_{P}-E_{M}\right)}^{3}}+\frac{1}{2}\underset{\mu \mu^{\prime }}{\sum }\underset{P\neq M,\pi }{\sum }\underset{N\neq M,\nu }{\sum }\beta \frac{\langle M\mu \left|\frac{\partial H}{\partial \lambda^{l}}\right|P\pi \rangle \left\langle P\pi \left|\frac{\partial H}{\partial \lambda^{k}}\right|M\mu^{\prime }\right\rangle \left\langle M\mu^{\prime }\left|\frac{\partial H}{\partial \lambda^{j}}\right|N\nu \right\rangle \left\langle N\nu \left|\frac{\partial H}{\partial \lambda^{i}}\right|M\mu \right\rangle }{\left(E_{N}-E_{M}\right)\left(E_{P}-E_{M}\right)}+\underset{\mu \mu^{\prime }}{\sum }\underset{P\neq M,\pi }{\sum }\underset{N\neq M,\nu }{\sum }\beta \frac{\langle M\mu \left|\frac{\partial H}{\partial \lambda^{l}}\right|P\pi \rangle \left\langle P\pi \left|\frac{\partial H}{\partial \lambda^{k}}\right|N\nu \right\rangle \left\langle N\nu \left|\frac{\partial H}{\partial \lambda^{j}}\right|M\mu^{\prime }\right\rangle \left\langle M\mu^{\prime }\left|\frac{\partial H}{\partial \lambda^{i}}\right|M\mu \right\rangle }{\left(E_{N}-E_{M}\right)\left(E_{P}-E_{M}\right)}-\underset{\mu \mu^{\prime }}{\sum }\underset{P\neq M,\pi }{\sum }\underset{N\neq M,\nu }{\sum }\frac{\langle M\mu \left|\frac{\partial H}{\partial \lambda^{l}}\right|P\pi \rangle \left\langle P\pi \left|\frac{\partial H}{\partial \lambda^{k}}\right|M\mu^{\prime }\right\rangle \left\langle M\mu^{\prime }\left|\frac{\partial H}{\partial \lambda^{j}}\right|N\nu \right\rangle \left\langle N\nu \left|\frac{\partial H}{\partial \lambda^{i}}\right|M\mu \right\rangle }{(E_{N}-E_{M}){\left(E_{P}-E_{M}\right)}^{2}}-\underset{\mu \mu^{\prime }}{\sum }\underset{P\neq M,\pi }{\sum }\underset{N\neq M,\nu }{\sum }\frac{\langle M\mu \left|\frac{\partial H}{\partial \lambda^{l}}\right|P\pi \rangle \left\langle P\pi \left|\frac{\partial H}{\partial \lambda^{k}}\right|N\nu \right\rangle \left\langle N\nu \left|\frac{\partial H}{\partial \lambda^{j}}\right|M\mu^{\prime }\right\rangle \left\langle M\mu^{\prime }\left|\frac{\partial H}{\partial \lambda^{i}}\right|M\mu \right\rangle }{(E_{P}-E_{M}){\left(E_{N}-E_{M}\right)}^{2}}-\underset{\mu \mu^{\prime }}{\sum }\underset{P\neq M,\pi }{\sum }\underset{N\neq M,\nu }{\sum }\frac{\langle M\mu \left|\frac{\partial H}{\partial \lambda^{l}}\right|P\pi \rangle \left\langle P\pi \left|\frac{\partial H}{\partial \lambda^{k}}\right|N\nu \right\rangle \left\langle N\nu \left|\frac{\partial H}{\partial \lambda^{j}}\right|M\mu^{\prime }\right\rangle \left\langle M\mu^{\prime }\left|\frac{\partial H}{\partial \lambda^{i}}\right|M\mu \right\rangle }{(E_{N}-E_{M}){\left(E_{P}-E_{M}\right)}^{2}}+\underset{\mu }{\sum }\underset{P\neq M,\pi }{\sum }\underset{N\neq M,\nu }{\sum }\underset{O\neq M,o}{\sum }\frac{\langle M\mu \left|\frac{\partial H}{\partial \lambda^{l}}\right|N\nu \rangle \left\langle N\nu \left|\frac{\partial H}{\partial \lambda^{k}}\right|\mathrm{Oo}\right\rangle \left\langle \mathrm{Oo}\left|\frac{\partial H}{\partial \lambda^{j}}\right|P\pi \right\rangle \left\langle P\pi \left|\frac{\partial H}{\partial \lambda^{i}}\right|M\mu \right\rangle }{\left(E_{N}-E_{M}\right)\left(E_{O}-E_{M}\right)\left(E_{P}-E_{M}\right)}\right]\right\}$$
129

Note that when the derivatives of the Helmholtz free energy are evaluated at zero field, the first and third derivatives are exactly zero.

### A.5 Response Function Notation

Since we neglected Hamiltonian derivatives of higher than first order, we can, in analogy to the derivative theory of the ground state energy, write the derivatives of the Helmholtz free energy as temperature-dependent generalizations of zero-frequency response functions,^45^ i.e.,

130
$$\frac{\partial^{2}F}{\partial \lambda^{i}\partial \lambda^{j}}=\left\langle \langle \frac{\partial H}{\partial \lambda^{i}}\frac{\partial H}{\partial \lambda^{j}}\rangle \right\rangle$$

131
$$\frac{\partial^{3}F}{\partial \lambda^{i}\partial \lambda^{j}\partial \lambda^{k}}=\left\langle \langle \frac{\partial H}{\partial \lambda^{i}}\frac{\partial H}{\partial \lambda^{j}}\frac{\partial H}{\partial \lambda^{k}}\rangle \right\rangle$$

132
$$\frac{\partial^{4}F}{\partial \lambda^{i}\partial \lambda^{j}\partial \lambda^{k}\partial \lambda^{l}}=\left\langle \langle \frac{\partial H}{\partial \lambda^{i}}\frac{\partial H}{\partial \lambda^{j}}\frac{\partial H}{\partial \lambda^{k}}\frac{\partial H}{\partial \lambda^{l}}\rangle \right\rangle$$

The advantage of this notation is that it is more general than derivatives of free energy, also allowing operators that are *not* derivatives of the Hamiltonian. From the sum-over-states expressions, it becomes clear that these “response functions” are linear in each of the operators. For example, in the spin Hamiltonian approximation, $\frac{\partial H}{\partial B_{0,i}}=\frac{1}{2}\sum_{j}g_{ij}S_{j}$ , i.e., the derivative of the Hamiltonian is a linear combination of spin operators. Because of the linearity of the “response function” in each operator, that means that we can, for example, write

133
$$\frac{\partial^{2}F}{\partial B_{0,i}\partial B_{0,j}}=\frac{1}{4}\sum_{\mathrm{kl}}g_{\mathrm{ik}}g_{\mathrm{jl}}\left\langle \left\langle S_{k}S_{l}\right\rangle \right\rangle$$

This means that the 2nd derivative of the Helmholtz free energy can be expressed in terms of a “spin dyadic”, i.e., a temperature-dependent linear response function of the spin operators.

## Notes

Description of the LFT implementation, experimental details, computational details of the quantum chemical calculations, convergence of finite-field shifts with REPULSION grids, deviation of second-order shifts from finite-field shifts at lower temperature (PDF)

## #

Max-Planck-Institut für Kohlenforschung, Kaiser-Wilhelm-Platz 1, 45470 Mülheim an der Ruhr, Germany
