# Supplementary material for: Theory of Field-Dependent NMR Shifts in Paramagnetic Molecules
Source: J Chem Theory Comput. 2025 May 27;21(11):5642–60. doi: 10.1021/acs.jctc.5c00433 (PMC12231273; doi:10.1021/acs.jctc.5c00433)
Supplement: Supplementary file 1 [file ct5c00433_si_001.pdf]

Supporting Information: Theory of Field-Dependent NMR Shifts in Paramagnetic Molecules

Lucas Lang,[*,†] Letizia Fiorucci,[‡,¶,§] Giacomo Parigi,[‡,¶] Claudio Luchinat,[‡,¶,‖] and Enrico Ravera[‡,¶,⊥]

†*Technische Universität Berlin, Institut für Chemie, Theoretische Chemie/Quantenchemie, Sekr. C7, Straße des 17. Juni 135, 10623 Berlin, Germany*

‡*Department of Chemistry "Ugo Schiff" and Magnetic Resonance Center (CERM), University of Florence, 50019 Florence, Italy*

¶*Consorzio Interuniversitario Risonanze Magnetiche di Metalloproteine (CIRMMP), 50019 Florence, Italy*

§*Present address: Max-Planck-Institut für Kohlenforschung, Kaiser-Wilhelm-Platz 1, 45470 Mülheim an der Ruhr, Germany*

‖*Giotto Biotech S.R.L, Sesto Fiorentino, 50019, Italy*

⊥*Florence Data Science, University of Florence, 50134 Florence, Italy*

E-mail: lucas.lang@chem.tu-berlin.de

S1 Implementation of LFT in `ParaMag.jl`

S1.1 LFT Hamiltonian in second quantization

In second quantization and using atomic units, the LFT Hamiltonian including SOC and the Zeeman effect is given by

$$H^{\mathrm{LFT}} = H^{\mathrm{LFT,\ nonrel}} + H^{\mathrm{SOC}} + H^{\mathrm{Zeeman}} \tag{S1}$$

and the individual parts are given by

$$H^{\mathrm{LFT,\ nonrel}} = \sum_{pq} [h^{\mathrm{LF}}_{pq} - \frac{1}{2}\sum_{r}(pr|rq)]E_{pq} + \frac{1}{2}\sum_{pqrs}(pq|rs)E_{pq}E_{rs} \tag{S2}$$

$$H^{\mathrm{SOC}} = \zeta \left[\frac{1}{2}\sum_{pq} l^{+}_{pq}s^{-}_{pq} + \frac{1}{2}\sum_{pq} l^{-}_{pq}s^{+}_{pq} + \sum_{pq} l^{z}_{pq}s^{z}_{pq} \right] \tag{S3}$$

$$H^{\mathrm{Zeeman}} = \frac{1}{2}\mathbf{B}\cdot\mathbf{L} + \mathbf{B}\cdot\mathbf{S} \tag{S4}$$

In the last equation,

$$L^{i} = \sum_{pq} l^{i}_{pq}E_{pq} \tag{S5}$$

$$S^{x} = \frac{1}{2}\left(\sum_{p} s^{+}_{pp} + \sum_{p} s^{-}_{pp}\right) \tag{S6}$$

$$S^{y} = \frac{1}{2i}\left(\sum_{p} s^{+}_{pp} - \sum_{p} s^{-}_{pp}\right) \tag{S7}$$

$$S^{z} = \sum_{p} s^{z}_{pp} \tag{S8}$$

The four basis single excitation operators are defined via

$$E^{\alpha}_{pq} = a^{\dagger}_{p\alpha} a_{q\alpha} \tag{S9}$$

$$E^{\beta}_{pq} = a^{\dagger}_{p\beta} a_{q\beta} \tag{S10}$$

$$s^{+}_{pq} = a^{\dagger}_{p\alpha} a_{q\beta} \tag{S11}$$

$$s^{-}_{pq} = a^{\dagger}_{p\beta} a_{q\alpha} \tag{S12}$$

From these, one can define two composite single excitation operators,

$$E_{pq} = E^{\alpha}_{pq} + E^{\beta}_{pq} \tag{S13}$$

$$s^{z}_{pq} = \frac{1}{2}(E^{\alpha}_{pq} - E^{\beta}_{pq}) \tag{S14}$$

S1.2 Parameters of the LFT Hamiltonian

The unique elements of the symmetric matrix h^{LF}_{pq} are all used as free parameters. I.e., there are 15 parameters for a d shell and 28 parameters for an f shell describing the ligand field. However, if there is symmetry, the number of free parameters is reduced. E.g., for a transition metal complex in an octahedral ligand field, only two parameters (the t_{2g} and the e_g orbital energies) occur. Since only their difference (the ligand field splitting Δ) is relevant for energy differences, an octahedral ligand field for a transition metal complex is parametrized only by a single parameter instead of 15.

The two-electron repulsion integrals (2ERIs) $(pq|rs)$ are parametrized through Slater–Condon parameters or Racah parameters, where one can safely set $F_0 = 0$ or $A = 0$and thereby reduce the number of electron repulsion parameters by one (because relative energies are not affected). SOC is parametrized through a single free parameter ζ and the Zeeman part does not contain any free parameters.

It remains to specify the orbital angular momentum integrals in the basis of d or f orbitals. The matrix elements of the operators $l^z, l^{\pm}$ in the basis of complex atomic-like

orbitals are given by

$$\langle m'|l^z|m\rangle = m\delta_{m'm} \tag{S15}$$

$$\langle m'|l^\pm|m\rangle = \sqrt{l(l+1)-m(m\pm 1)}\delta_{m',m\pm 1} \tag{S16}$$

S1.3 Labeling of orbitals and Slater determinants

For N electrons in M spin orbitals ($M = 10$ for a d shell and $M = 14$ for an f shell), there are $\binom{M}{N}$ Slater determinants. They comprise the so-called full configuration interaction (FCI) space. Each spin orbital can either be characterized by a spin orbital label P or a spatial orbital label p together with specification of the spin part σ (α or β). We chose the order of spin orbitals as $1\alpha, 1\beta, 2\alpha, 2\beta, \ldots$, such that one can convert between the two labelings as follows:

$$P_{p\sigma} = \begin{cases} 2p-1 & \text{if } \sigma = \alpha \\ 2p & \text{if } \sigma = \beta \end{cases} \tag{S17}$$

$$p_P = (P+1) \div 2 \tag{S18}$$

$$\sigma_P = \begin{cases} \alpha & \text{if } (P+1)\%2 = 0 \\ \beta & \text{if } (P+1)\%2 = 1 \end{cases} \tag{S19}$$

Here, $\div$ denotes integer division and % the remainder (modulo operation). For both spin orbital (P) and spatial orbital (p) labels, we start counting at 1 as is usual in Julia.

Slater determinants are labeled by strings of spin orbital labels. E.g., $|123\rangle = |1\alpha 1\beta 2\alpha\rangle$ is a Slater determinant that has spatial orbital 1 doubly occupied and spatial orbital 2 singly occupied with α spin. By convention, the spin orbital labels are always ordered within the orbital string. For implementation purposes, it is furthermore necessary to label the Slater determinants with a single contiguous integer index running from 1 to $\binom{M}{N}$. In order to do so, the strings of spin orbital labels are lexicographically ordered. For example, if there are

$N = 3$ electrons in $M = 5$ spin orbitals, the 10 resulting Slater determinants are ordered as $|123\rangle, |124\rangle, |125\rangle, |134\rangle, |135\rangle, |145\rangle, |234\rangle, |235\rangle, |245\rangle, |345\rangle$. Then, e.g., the index of the determinant $|134\rangle$ is $I = 4$, and we write $|I = 4\rangle = |134\rangle$.

S1.4 Excitation generation

For each determinant $|K\rangle$, we first generate four lists: The occupied α spin orbitals, occupied β spin orbitals, unoccupied α spin orbitals, and unoccupied β spin orbitals. For the two lists of occupied orbitals, we also save the position (electron index) of each orbital within the orbital string of $|K\rangle$. Next, we generate four lists of excitations by combining possible occupied with possible unoccupied orbitals:

- For excitations of occ. α to unocc. α or to itself: store I, p, q, $\gamma = \langle I|E^{\alpha}_{pq}|K\rangle$ in the list L^{α}_{K}
- For excitations of occ. α to unocc. β: store I, p, q, $\gamma = \langle I|s^{-}_{pq}|K\rangle$ in the list L^{-}_{K}
- For excitations of occ. β to unocc. β or to itself: store I, p, q, $\gamma = \langle I|E^{\beta}_{pq}|K\rangle$ in the list L^{β}_{K}
- For excitations of occ. β to unocc. α: store I, p, q, $\gamma = \langle I|s^{+}_{pq}|K\rangle$ in the list L^{+}_{K}

An individual excitation can be generated as follows: For a given occupied spin orbital Q with spatial label q and unoccupied (or same) spin orbital P with spatial label p, we first take the orbital string of $|K\rangle$ (using spin orbital labels), and replace Q in the correct (previously saved) position by P. Then, we sort/order the resulting string. The coupling coefficient γ is equal to the sign of the sorting permutation. The index of the generated determinant can be calculated using equations given by Knowles and Handy in their discussion of the nonrelativistic full CI method:[1,2] the index can be calculated as

$$I = 1 + \sum_{i=1}^{N} Z(i, P_i) \tag{S20}$$

with

$$Z(i,P) = \begin{cases} \sum_{m=M-P+1}^{M-i} \left[\binom{m}{N-i} - \binom{m-1}{N-i-1}\right] & \text{if } i < N \\ P - N & \text{if } i = N \end{cases} \tag{S21}$$

where P_i is the index of the spin orbital in (electron) position i of the spin orbital string obtained after excitation and ordering. It should be noted that Knowles and Handy applied these equations to find separate indices for α and β strings of spatial orbitals, while we apply them for finding indices of complete Slater determinants containing both α and β orbitals.

For easy calculation of the total spin operator matrix elements, one can store the excitations between different spatial orbitals ($p \neq q$) separate from the excitations between the same spatial orbitals ($p = q$), or at least save the indices of each type of excitation within the total list. We will refer to the list containing only excitations between equal spatial orbitals as $L_K^{\alpha,\text{same}}$, and similarly for the three other lists. As an alternative to this procedure is using identity matrices (Kronecker deltas) as "integrals" that are contracted with the coupling coefficients in the calculation of the spin operators. This is less computationally efficient, but in the case of LFT, where the full CI space is very small, this probably does not matter much.

S1.5 Calculation of the Hamiltonian matrix

S1.5.1 Nonrelativistic Hamiltonian

Single-excitation part Defining the abbreviation

$$\tilde{h}_{pq} = h_{pq}^{\text{LF}} - \frac{1}{2}\sum_r (pr|rq) \tag{S22}$$

the single-excitation contribution to the nonrelativistic Hamiltonian is

$$\langle I|H^{\text{LFT, nonrel(1)}}|J\rangle = \sum_{pq} \tilde{h}_{pq}\langle I|E_{pq}|J\rangle \tag{S23}$$

This matrix can be calculated via algorithm 1.

Algorithm 1 Calculation of the single-excitation part of the nonrelativistic LFT Hamiltonian.

for all determinants $|J\rangle$ **do**
    for $i \in L_J^\alpha$ **do**
        $\langle I_i|H^{\text{LFT, nonrel(1)}}|J\rangle \mathrel{+}= \tilde{h}_{p_i q_i}\gamma_i$
    end for
    for $i \in L_J^\beta$ **do**
        $\langle I_i|H^{\text{LFT, nonrel(1)}}|J\rangle \mathrel{+}= \tilde{h}_{p_i q_i}\gamma_i$
    end for
end for

Double-excitation part The matrix representation of the double-excitation part of the nonrelativistic Hamiltonian can be written as

$$\langle I|H^{\text{LFT, nonrel(2)}}|J\rangle = \frac{1}{2}\sum_K\sum_{pq}\langle I|E_{pq}|K\rangle X_{pq}^J \tag{S24}$$

where for implementation purposes, we define the intermediate

$$X_{pq}^J = \sum_{rs}(pq|rs)\langle K|E_{rs}|J\rangle \tag{S25}$$

This intermediate is specific to a given determinant $|K\rangle$. However, it is only calculated for one $|K\rangle$ at a time. Hence, we drop the K label.

The double-excitation part of the nonrelativistic LFT Hamiltonian can be calculated via algorithm 2.

S1.5.2 Spin–orbit Hamiltonian

The matrix representation of the SOC Hamiltonian in the basis of Slater determinants can be written as

$$\langle I|H^{\text{SOC}}|J\rangle = \zeta\left[\frac{1}{2}\sum_{pq} l_{pq}^{+}\langle I|s_{pq}^{-}|J\rangle + \frac{1}{2}\sum_{pq} l_{pq}^{-}\langle I|s_{pq}^{+}|J\rangle + \sum_{pq} l_{pq}^{z}\langle I|s_{pq}^{z}|J\rangle\right] \tag{S26}$$

Algorithm 2 Calculation of the double-excitation part of the nonrelativistic LFT Hamiltonian.

for all determinants $|K\rangle$ **do**
  Initialize X^{I}_{pq} to 0
  for all orbital pairs p, q **do**
    for $i \in L^{\alpha}_{K}$ **do**
      $X^{I_i}_{pq} \mathrel{+}= (pq|q_i p_i)\gamma_i$
    end for
    for $i \in L^{\beta}_{K}$ **do**
      $X^{I_i}_{pq} \mathrel{+}= (pq|q_i p_i)\gamma_i$
    end for
  end for
  for all determinants $|J\rangle$ **do**
    for $i \in L^{\alpha}_{K}$ **do**
      $\langle I_i|H^{\text{LFT, nonrel(2)}}|J\rangle \mathrel{+}= \frac{1}{2}\gamma_i X^{J}_{p_i q_i}$
    end for
    for $i \in L^{\beta}_{K}$ **do**
      $\langle I_i|H^{\text{LFT, nonrel(2)}}|J\rangle \mathrel{+}= \frac{1}{2}\gamma_i X^{J}_{p_i q_i}$
    end for
  end for
end for

This matrix can be calculated via algorithm 3. Note that in the very last loop, the contributions are *subtracted from* instead of added to the Hamiltonian matrix.

S1.5.3 Total orbital angular momentum operators

The matrix representation of total orbital angular momentum operators in the basis of Slater determinants can be written as

$$\langle I|L^k|J\rangle = \sum_{pq} l^k_{pq} \langle I|E_{pq}|J\rangle \tag{S27}$$

This matrix can be calculated via algorithm 4, which is essentially the same as the algorithm for calculation of the single-excitation contribution to the nonrelativistic Hamiltonian.

Algorithm 3 Calculation of the SOC Hamiltonian.

for all determinants $|J\rangle$ **do**
    for $i \in L_J^-$ **do**
        $\langle I_i|H^{\mathrm{SOC}}|J\rangle \mathrel{+}= \zeta \frac{1}{2} l^+_{p_i q_i} \gamma_i$
    end for
    for $i \in L_J^+$ **do**
        $\langle I_i|H^{\mathrm{SOC}}|J\rangle \mathrel{+}= \zeta \frac{1}{2} l^-_{p_i q_i} \gamma_i$
    end for
    for $i \in L_J^\alpha$ **do**
        $\langle I_i|H^{\mathrm{SOC}}|J\rangle \mathrel{+}= \zeta \frac{1}{2} l^z_{p_i q_i} \gamma_i$
    end for
    for $i \in L_J^\beta$ **do**
        $\langle I_i|H^{\mathrm{SOC}}|J\rangle \mathrel{-}= \zeta \frac{1}{2} l^z_{p_i q_i} \gamma_i$
    end for
end for

Algorithm 4 Calculation of the total orbital angular momentum matrices.

for all determinants $|J\rangle$ **do**
    for $i \in L_J^\alpha$ **do**
        $\langle I_i|L^k|J\rangle \mathrel{+}= l^k_{p_i q_i} \gamma_i$
    end for
    for $i \in L_J^\beta$ **do**
        $\langle I_i|L^k|J\rangle \mathrel{+}= l^k_{p_i q_i} \gamma_i$
    end for
end for

S1.5.4 Total spin operators

The matrix representation of the total spin operators can be written as

$$\langle I|S^x|J\rangle = \frac{1}{2}\left(\sum_{pq}\delta_{pq}s^{+}_{pq} + \sum_{pq}\delta_{pq}s^{-}_{pq}\right) \tag{S28}$$

$$\langle I|S^y|J\rangle = \frac{1}{2i}\left(\sum_{pq}\delta_{pq}s^{+}_{pq} - \sum_{pq}\delta_{pq}s^{-}_{pq}\right) \tag{S29}$$

$$\langle I|S^z|J\rangle = \sum_{pq}\delta_{pq}s^{z}_{pq} \tag{S30}$$

Note that as mentioned above the explicit use of identity matrix "integrals" makes the computation more expensive (which does not matter for our purposes) while making the implementation easier (because the same full excitation lists can be used as for the other operators).

The calculation of these operators is shown in algorithm 5. Note again the cases where the contribution is subtracted from, instead of added to, the corresponding matrix element.

Algorithm 5 Calculation of the total spin matrices.

for all determinants $|J\rangle$ **do**
  for $i \in L^{+}_{J}$ **do**
    $\langle I_i|S^x|J\rangle \mathrel{+}= \frac{1}{2}\delta_{p_i q_i}\gamma_i$
    $\langle I_i|S^y|J\rangle \mathrel{+}= \frac{1}{2i}\delta_{p_i q_i}\gamma_i$
  end for
  for $i \in L^{-}_{J}$ **do**
    $\langle I_i|S^x|J\rangle \mathrel{+}= \frac{1}{2}\delta_{p_i q_i}\gamma_i$
    $\langle I_i|S^y|J\rangle \mathrel{-}= \frac{1}{2i}\delta_{p_i q_i}\gamma_i$
  end for
  for $i \in L^{\alpha}_{J}$ **do**
    $\langle I_i|S^z|J\rangle \mathrel{+}= \frac{1}{2}\delta_{p_i q_i}\gamma_i$
  end for
  for $i \in L^{\beta}_{J}$ **do**
    $\langle I_i|S^z|J\rangle \mathrel{-}= \frac{1}{2}\delta_{p_i q_i}\gamma_i$
  end for
end for

S2 Extraction of experimental hyperfine shifts

The NiSAL-HDPT complex was synthesized as described in.[3] The sample was composed of a 5 mM NiSAL-HDPT solution in deuterated chloroform. The experimental spectra were acquired on a Bruker Avance III spectrometer operating at 400 MHz ^{1}H Larmor frequency (9.4 T) equipped with a 5 mm ^{1}H selective probe dedicated to paramagnetic systems, and on a Bruker Avance NEO spectrometer operating at 1.2 GHz ^{1}H Larmor frequency with a 28.2 T HTS/LTS hybrid magnet, using a 3 mm triple resonance TCI cryo-probehead. The NMR data were acquired using Bruker TopSpin v4.0.6 software. A temperature calibration with a deuterated methanol 99.8% standard sample was performed before each acquisition session and the acquisition temperature was set to 298.0 K both at high and low field, with an uncertainty of about $\pm$ 0.05 K (see Figures S1 and S2).

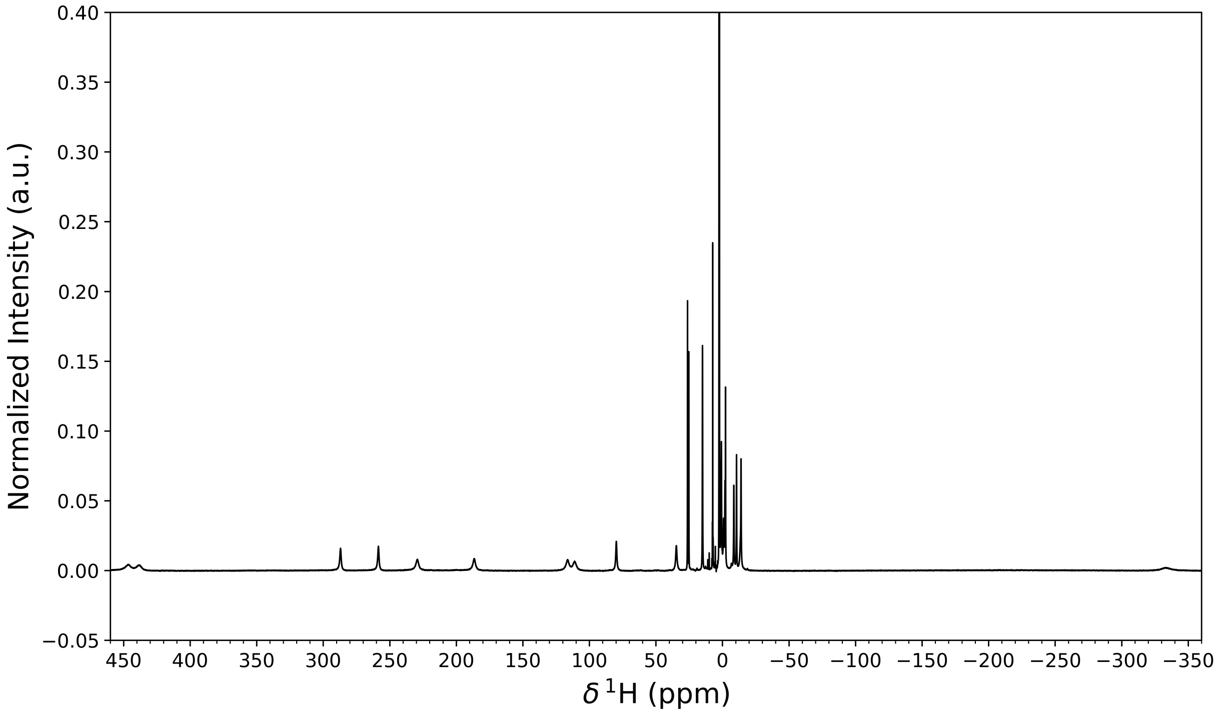

Figure S1: Experimental spectrum of NiSAL-HDPT acquired at 400 MHz ^{1}H Larmor frequency and 298.0 K.

The experimental shifts were extracted through an automated fitting procedure, where

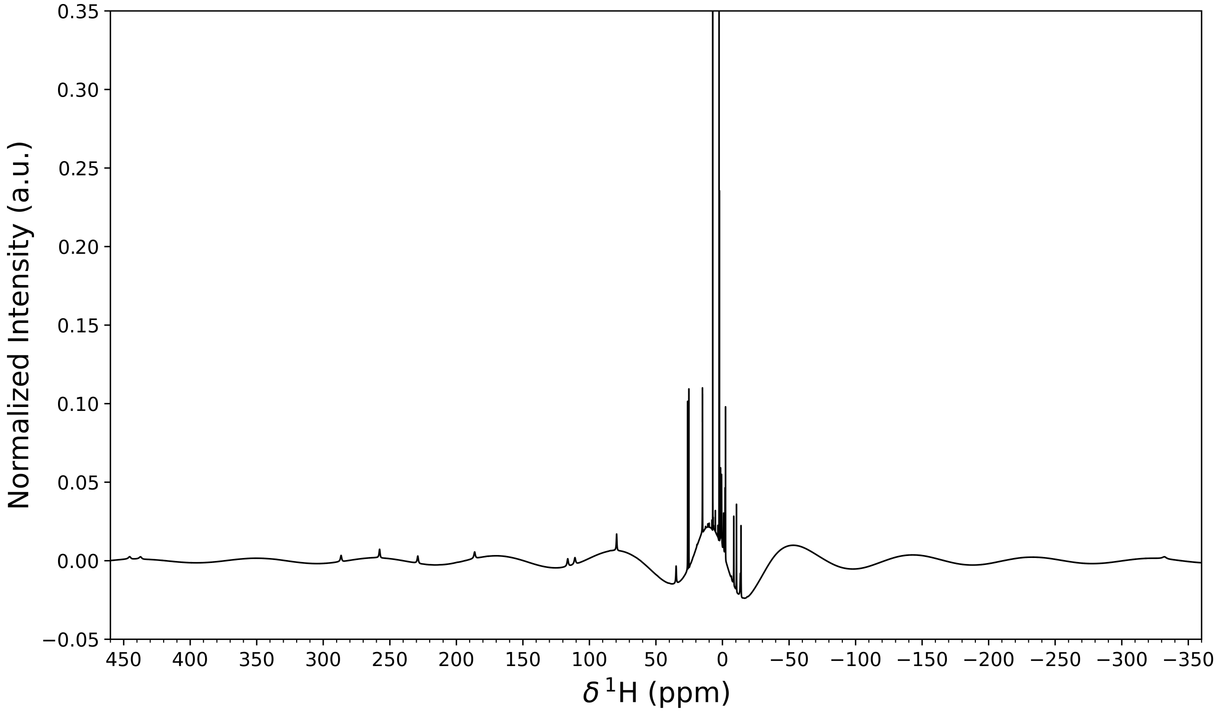

Figure S2: Experimental spectrum of NiSAL-HDPT acquired at 1.2 GHz ^{1}H Larmor frequency and 298.0 K.

the paramagnetic signals were modeled using a Voigt lineshape and a polynomial function as baseline. The routines, implemented in Python, are available in a GitHub repository (`https://github.com/letiziafiorucci/tragico`).

Not all the peaks (especially those in the diamagnetic region) were perfectly isolated due to strong overlap. Furthermore, the smaller the magnitude of the shift, the smaller is also the magnitude of the field-dependent part $\Delta\delta$. Therefore, the comparison with the simulated counterpart was performed on a subset of the experimental shifts, i.e., those for which the expected field-dependent correction is sensibly higher than the error from the peak's deconvolution method and the plausible temperature-related uncertainty (see Table S1).

Table S1: Experimental shifts extracted from the spectra acquired at 298.0 K for NiSAL-HDPT and experimental field dependence, $\Delta\delta = \delta(1.2\ \text{GHz}) - \delta(400\ \text{MHz})$ (both in ppm) and $\Delta\delta^{\text{rel}} = |\Delta\delta/\delta(400\ \text{MHz})| \times 100$ (in %). The absolute error (ε) in the shift values is estimated from the fitting procedure. The assignment is taken from.[4]
(*)Peaks are not considered in the comparison with simulated shifts due to complete overlap with others or too small variation.

label	δ_{exp} (400 MHz)	ε	δ_{exp} (1.2 GHz)	ε	$\Delta\delta_{\text{exp}}$	$\Delta\delta_{\text{exp}}^{\text{rel}}$
NH	−333.074	0.107	−332.250	0.065	0.824	0.247
3	−13.996	0.004	−13.980	0.004	0.016	0.114
3’	−13.535	0.019	−13.502	0.005	0.033	0.244
β_1’	−10.609	0.001	−10.584	0.001	0.025	0.236
β_2’	−8.579	0.003	−8.551	0.003	0.028	0.326
5’(*)	−2.340	0.007	−2.343	0.002	−0.003	−0.128
β_1(*)	−1.907	0.044	−1.965	0.000	−0.058	−3.041
β_2(*)	−0.894	0.189	−0.886	0.001	0.008	0.895
5(*)	2.552	0.001	2.524	–	−0.028	1.097
6’(*)	14.909	–	14.925	0.003	0.016	−0.107
6(*)	15.008	–	15.007	0.002	−0.001	0.007
4’	25.246	0.004	25.193	0.004	−0.053	0.210
4	26.214	0.004	26.159	0.004	−0.055	0.210
α_1’(*)	34.631	0.012	34.780	0.010	0.149	−0.430
α_2	79.710	0.011	79.479	0.010	−0.231	0.290
γ_2’	110.991	0.034	110.799	–	−0.192	0.173
γ_2	116.320	0.030	116.209	–	−0.111	0.095
γ_1’	186.455	0.040	186.163	0.036	−0.292	0.157
γ_1	229.201	0.045	228.880	0.040	−0.321	0.140
α_2’	258.472	0.047	257.578	0.046	−0.894	0.346
α_1	286.931	0.048	286.490	0.047	−0.441	0.154
CH	438.033	0.100	437.152	0.079	−0.881	0.201
CH’	446.273	0.098	445.287	0.078	−0.986	0.221

S3 Computational details for the quantum-chemical calculation of spin Hamiltonian and LFT parameters

The geometry of NiSAL-HDPT used here is the structure that gave the best agreement between calculated and experimental shifts in our previous work.[4]

For the calculation of the zero-field splitting **D**-tensor and the LFT parameters via AILFT, we performed a CASSCF calculation with 8 electrons in 5 active orbitals, state-averaged over 10 triplet and 15 singlet roots. Scalar relativity was described at the DKH2 level using the DKH-def2-TZVP basis set and the RI approximation using an AutoAux auxiliary basis set. On top, we performed SC-NEVPT2 with all orbitals correlated (no frozen core). The subsequent QDPT calculation with NEVPT2 diagonal energies employed the SOMF effective one-electron spin–orbit coupling operator with one-center exchange.

For obtaining the A_{iso} values and the **g**-tensor, we performed a DFT calculation with B3LYP, DKH2 scalar relativity, DKH-def2-TZVP basis set, RIJCOSX approximation with def2/J auxiliary basis set and DEFGRID3 numerical integration grid as well as TightSCF convergence settings. The **g**-tensor consists of the free electron g_{e} value plus contributions that depend only on the spin density (relativistic mass correction and gauge correction) as well as the mixed orbital Zeeman / SOC contribution calculated via CP-SCF. The A_{iso} values are dominated by the Fermi contact contribution (which depends on the spin density), but are also affected by the mixed orbital / SOC contribution calculated via CP-SCF. SOC was described via the SOMF operator with one-center exchange.

For more details, see the complete ORCA output files that are also provided by us.

S4 Convergence of the finite field shifts using repulsion grids

Figure S3 shows the convergence of the finite field shifts with respect to increasing the grid size for REPULSION[5] grids instead of the Lebedev grids used in the main paper. We took the grids from the SIMPSON package,[6] whose source code is available at `https://github.com/vosegaard/simpson`. The grid points of the largest REPULSION grid (with 2000 grid points) have a weight of 5×10^{-4}, whereas the largest grid weight for the largest Lebedev grid we implemented (having 5810 grid points) is $\approx 1.9 \times 10^{-4}$, which is smaller. Therefore, we used the largest Lebedev grid as reference because we expect it to be more accurate than the largest REPULSION grid.

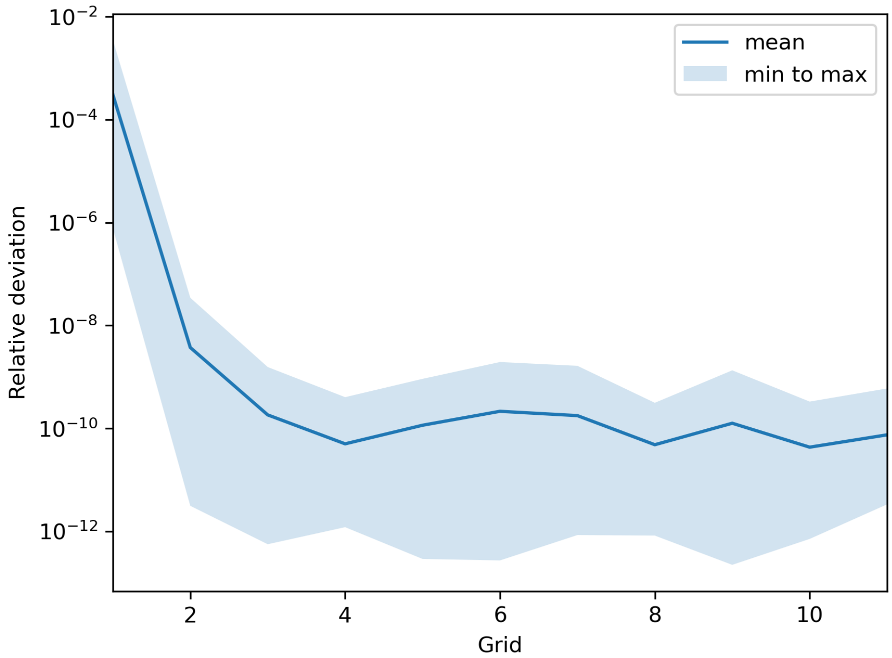

Figure S3: Convergence of the finite field shifts (relative absolute deviation from reference grid, see main paper) as a function of the grid at 400 MHz ^{1}H Larmor frequency and 298 K. Here, we use the REPULSION grids from the SIMPSON package[6] with sizes of 10, 20, 30, 66, 100, 144, 168, 256, 320, 678, 2000 points.

S5 Comparison of 2nd order and finite field shifts at lower temperature

In the main manuscript, we compare 2nd order and finite field shifts at room temperature (298 K). It is expected that the 2nd order approach becomes a worse approximation for lower temperatures. Therefore, in Figure S4 we show a similar plot for a lower temperature of 223 K (≈ -50 °C). It can be seen that the errors of the 2nd order approach are slightly larger than at room temperature but still in the same order of magnitude, meaning that the 2nd order approach is still applicable for routinely accessible field strengths also at this lower temperature.

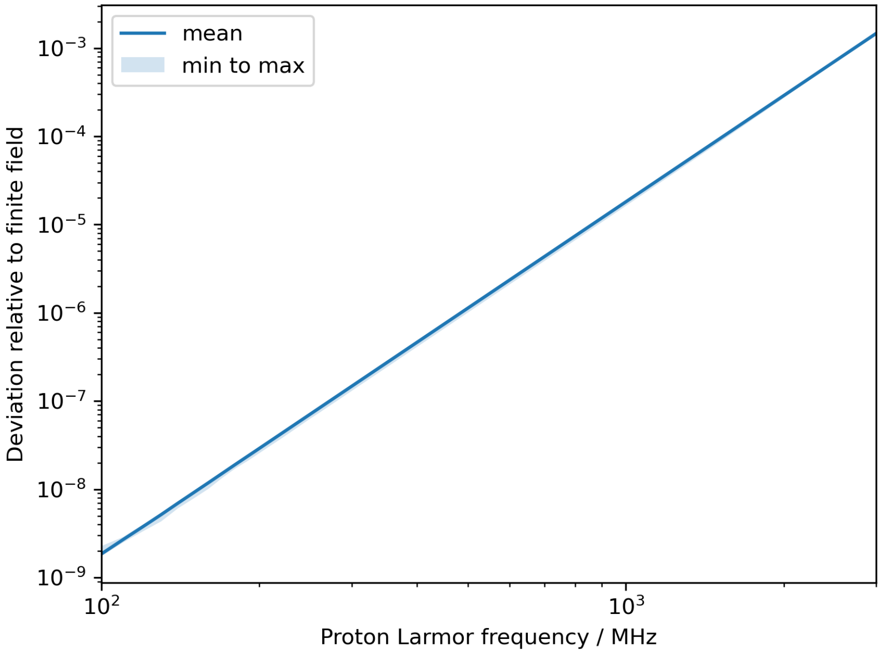

Figure S4: Relative absolute deviation of 2nd order shifts from finite field shifts as a function of the field strength (measured in proton Larmor frequency).

References

(1) Knowles, P. J.; Handy, N. C. A new determinant-based full configuration interaction method. *Chem. Phys. Lett.* **1984**, *111*, 315–321.

(2) Knowles, P. J.; Handy, N. C. A determinant based full configuration interaction program. *Comput. Phys. Commun.* **1989**, *54*, 75–83.

(3) Sacconi, L.; Bertini, I. High-spin five-coordinated 3d metal complexes with pentadentate Schiff bases. *J. Am. Chem. Soc.* **1966**, *88*, 5180–5185.

(4) Ravera, E.; Gigli, L.; Czarniecki, B.; Lang, L.; Kümmerle, R.; Parigi, G.; Piccioli, M.; Neese, F.; Luchinat, C. A Quantum Chemistry View on Two Archetypical Paramagnetic Pentacoordinate Nickel(II) Complexes Offers a Fresh Look on Their NMR Spectra. *Inorg. Chem.* **2021**, *60*, 2068–2075.

(5) Bak, M.; Nielsen, N. C. REPULSION, A Novel Approach to Efficient Powder Averaging in Solid-State NMR. *J. Magn. Reson.* **1997**, *125*, 132–139.

(6) Bak, M.; Rasmussen, J. T.; Nielsen, N. C. SIMPSON: A general simulation program for solid-state NMR spectroscopy. *J. Magn. Reson.* **2000**, *213*, 366–400.
